# From Signal to Memory: A Proposed Adaptive Hierarchy Linking Autism-Associated Genes to Sensory Homeostasis

**DOI:** 10.3390/genes17101273

**Published:** 2026-10-13

**Authors:** John Jay Gargus

**Affiliations:** 1 Departments of Pediatrics, Medical Genetics & Genomics, Physiology & Biophysics and Center for Autism Research & Translation, University of California, Irvine, CA 92697, USA; jjgargus@uci.edu; 2 NeuroQure, Dana Point, CA 92629, USA

**Keywords:** autism spectrum disorder, sensory homeostasis, sensory processing, intracellular calcium signaling, inositol 1,4,5-trisphosphate receptor, activity-dependent transcription, chromatin regulation, adaptive cellular memory

## Abstract

Every nervous system must preserve sensitivity to change while reducing responses to constancy. This review defines that adaptive objective as *sensory homeostasis*: the process through which familiar information recedes while unexpected or biologically important events remain salient. Its disruption is particularly relevant to autism spectrum disorder (ASD), in which sensory abnormalities occur across multiple modalities. Large-scale sequencing studies have identified hundreds of ASD-associated genes spanning neuronal excitability, synaptic function, intracellular signaling, transcription, and chromatin regulation. This hypothesis-driven narrative review asks whether the established functions of independently identified ASD-associated genes support a directional framework through which genetic vulnerability could compromise sensory homeostasis. Beginning with a prespecified Autism Sequencing Consortium-derived set of 185 ASD-associated genes, and separately classifying complementary genetic and mechanistic evidence, the review organizes a bounded subset into three functional levels: generation and spatial organization of intracellular Ca^2+^ signals; interpretation of those signals by electrical, biochemical, synaptic, and transcriptional decoders; and stabilization of selected responses through transcriptional and chromatin-regulatory mechanisms that establish adaptive cellular memory. The resulting 44-gene core contains 32 of the 185 members distributed across all three levels and identifies IP_3_R-mediated endoplasmic-reticulum Ca^2+^ release as an important, but not exclusive, Level 1 convergence mechanism in signal generation. The proposed framework predicts distinct proximal defects that converge on abnormal response trajectories across repeated stimulation, providing a testable connection among ASD genetics, adaptive cellular memory, and sensory phenotype.

## 1. Introduction

Every nervous system must preserve sensitivity to change while reducing responses to constancy. The pressure of clothing, background ventilation, familiar visual structure, and persistent odors ordinarily recede, whereas unexpected or biologically important events retain salience. This review defines that adaptive objective as *sensory homeostasis*: the coordinated regulation of responsiveness through which repeated or predictable input becomes less salient while sensitivity to novel, changing, or biologically important input is preserved. Sensory homeostasis is an integrative outcome rather than a single mechanism. Habituation describes reduced responding during repeated stimulation; sensory adaptation describes changes produced by sustained or repeated input; gain control rescales neuronal input-output relationships; homeostatic plasticity stabilizes activity around a functional range; and predictive processing uses prior expectations to interpret incoming information. Sensory homeostasis can draw upon each of these processes but is defined here by their shared outcome: experience appropriately recalibrates subsequent responsiveness without eliminating sensitivity to meaningful change [1,2,3,4,5,6].

Sensory homeostasis requires a memory of prior activity. Neuronal stimulation must do more than produce an immediate electrical or biochemical response; it must modify how the cell responds when stimulation recurs. Transient activity can produce persistent but reversible changes in protein localization or abundance, receptor and ion-channel function, synaptic organization, transcription, chromatin accessibility, metabolism, and cellular structure [7,8,9,10,11]. The term *adaptive cellular memory* is used here for an activity-dependent change in cellular state that alters a later response. It does not denote behavioral learning, conscious recollection, or cognitive memory.

This adaptive problem is particularly relevant to autism spectrum disorder (ASD), a heterogeneous neurodevelopmental condition defined by persistent differences in social communication and social interaction together with restricted or repetitive patterns of behavior, interests, or activities. Hyperreactivity or hyporeactivity to sensory input and unusual interest in sensory aspects of the environment are included within the restricted and repetitive behavior domain, although sensory manifestations vary substantially among individuals [12]. Sensory hyperresponsiveness, hyporesponsiveness, and sensory seeking occur across multiple modalities and have been related to peripheral encoding, synaptic transmission, excitation-inhibition balance, circuit gain, attention, and predictive processing [13,14,15,16,17,18,19]. These mechanisms are not mutually exclusive, but cross-modal abnormalities could also arise from disruption of intracellular processes broadly used to convert sensory activity into adaptive change. Twin, family, and population studies indicate a substantial heritable contribution to ASD, commonly estimated at approximately 80–90%, although estimates vary with design and population [20]. This review uses neutral, respectful language while recognizing that preferences for identity-first and person-first terminology differ among autistic people and their families.

Large-scale genetic studies have identified ASD-associated genes spanning neuronal excitability, synaptic function, intracellular signaling, transcription, and chromatin regulation [21,22,23,24,25,26]. These categories organize an extensive gene list, but they are commonly presented as parallel contributors to neurodevelopmental pathology. Functional enrichment alone does not determine whether they occupy successive positions in a directional process or identify the adaptive function that might be impaired when that process is disrupted.

Intracellular Ca^2+^ signaling provides a biologically plausible focus for examining such convergence. Ca^2+^ signals connect membrane excitability and synaptic transmission to biochemical regulation, synaptic plasticity, transcription, chromatin state, metabolism, and subsequent cellular responsiveness. Their biological consequences depend on amplitude, duration, frequency, source, subcellular localization, and coupling to downstream decoders [27,28,29,30,31,32,33,34,35]. Disruption at different positions in this distributed system could therefore produce distinct proximal defects while converging on impaired activity-dependent recalibration. This rationale does not imply that Ca^2+^ signaling is the predominant pathway in ASD or that every ASD-associated gene participates in it; it defines a specific mechanistic hypothesis that can be evaluated against independently derived genetic evidence.

The primary genetic starting set is the 185-gene cohort identified by Fu et al. in 2022 (**Fu-185**), which extended the earlier Autism Sequencing Consortium analysis reported by Satterstrom et al. in 2020 (**Satterstrom-102**) [23,24]. Membership in either statistically defined set is termed ***ASC-core*** *genetic evidence*, while membership in the individual sets is distinguished where relevant. Neither set was selected for intracellular Ca^2+^ signaling, IP_3_-receptor biology, sensory function, or the signal-to-memory architecture proposed here. The analysis therefore asks whether the established functions of independently identified ASD-associated genes support a prespecified mechanistic framework; it does not claim that the framework emerged from an unbiased analysis of the complete Fu-185.

The proposed signal-to-memory (**S2M**) framework comprises three functional levels. Level 1 generates and spatially organizes activity-dependent Ca^2+^ *signals* through membrane excitability, synaptic transmission, Ca^2+^ entry, phosphoinositide signaling, molecular scaffolding, and intracellular Ca^2+^ release. Level 2 contains electrical, biochemical, synaptic, metabolic, and transcriptional *decoders* that interpret the magnitude, timing, and location of those signals. Level 3 contains transcriptional and chromatin-regulatory mechanisms that help stabilize selected consequences as a longer-lasting *memory* via changes in cellular state. Modified future responsiveness is the proposed functional output:

*Neuronal and sensory activity → Ca* ^2+^ *-signal generation → Ca* ^2+^ *-dependent decoding → transcriptional and chromatin memory → modified future responsiveness*

Within Level 1, phospholipase C-dependent production of inositol 1,4,5-trisphosphate (IP_3_) activates IP_3_ receptors (IP_3_Rs) in the endoplasmic reticulum [27,28,29,30]. IP_3_Rs provide an important, but not exclusive, convergence mechanism because their opening is shaped by IP_3_, cytosolic and luminal Ca^2+^, ATP and other nucleotides, phosphorylation, interacting proteins, and signaling feedback [28,30,31]. The resulting local, oscillatory, and propagating Ca^2+^ signals distribute activity-dependent information among synaptic, nuclear, cytoskeletal, mitochondrial, and other cellular compartments [29,31,32,33,34,35]. NMDA receptors, voltage-gated Ca^2+^ channels, ryanodine receptors, store-operated entry, mitochondrial handling, and plasma-membrane extrusion remain integral components of the broader Ca^2+^-signaling network.

The framework is directional but recurrent. Transcriptional and chromatin changes can alter the abundance or organization of receptors, channels, signaling proteins, synapses, metabolic machinery, and Ca^2+^ decoders, making the endpoint of one adaptive cycle part of the starting condition for the next [5,7,8,9,10,11,36].

Genetic association and mechanistic placement must be reported separately. *ITPR3* belongs to the Fu-185, whereas *ITPR1* does not belong to the ASC-core set but carries complementary ASD and neurodevelopmental evidence [23,24,37,38]. Experiments in the BTBR *T+ Itpr3*^tf^/J mouse further show that disruption of IP_3_R3-mediated Ca^2+^ release can impair responses to several receptor-distinct stimuli that share a common intracellular signal [39,40,41]. These observations motivate, but do not establish, the broader S2M framework.

This hypothesis-driven narrative review and structured mechanistic synthesis evaluates whether the established functions of a bounded subset of ASD-associated genes support a directional route through which neuronal activity could modify future cellular responsiveness. It distinguishes ASC-core genetic evidence, complementary genetic evidence, and mechanistic support; assigns genes to principal functional positions using explicit criteria; and derives predictions testable through repeated-stimulation paradigms. S2M is presented as a bounded and falsifiable framework, not as an obligatory linear pathway, an unbiased reconstruction of the Fu-185, or a comprehensive explanation of ASD genetics or sensory dysfunction.

## 2. Review Design, Gene Set, and Evidence Grading

### 2.1. Review Design and Literature Identification

S2M was developed as a structured mechanistic synthesis rather than as a systematic review or unsupervised pathway-enrichment analysis. The prespecified question was whether the established functions of genes identified independently through large-scale ASD sequencing support a directional sequence through which neuronal activity could modify future cellular responsiveness. Because the review did not seek exhaustive retrieval of all publications meeting study-level eligibility criteria, individual articles were not treated as units of inclusion in a systematic-review process, and PRISMA procedures were not applied.

Supporting literature was identified through targeted searches of PubMed and Google Scholar updated through September 2026. Searches combined each gene symbol, protein name, or recognized alias with terms relevant to ASD, neurodevelopmental disorders, neuronal activity, Ca^2+^ signaling, IP_3_ receptors, excitability, synaptic transmission, phosphoinositide signaling, calmodulin, kinases, phosphatases, transcription, chromatin regulation, mitochondria, adaptation, and plasticity. Reference lists of major reviews and primary studies were also examined.

Primary experimental studies were prioritized for gene-specific mechanistic assignments, large-scale sequencing studies for ASD-genetic evidence, and clinical genetic studies for established neurodevelopmental disorders. Authoritative reviews supported established biochemical principles and helped identify foundational experiments. A study was considered relevant when it established a molecular operation, localization, interaction, regulatory relationship, or physiological consequence bearing directly on the proposed position of a gene. General involvement in neurodevelopment, transcription, or chromatin regulation was insufficient for core placement.

Each retained claim was checked against its cited source for the gene or protein studied, experimental system, molecular relationship, and reported direction of effect. Evidence from human cells, animal models, invertebrate systems, or non-neuronal preparations was interpreted within the limitations of that system. Bibliographic details were verified against PubMed, publisher records, or Google Scholar, and gene symbols were normalized before final counts were calculated. Because the evidence spans heterogeneous designs, no single numerical quality score or formal risk-of-bias assessment was applied. Evidence was instead judged by relevance to the claim, directness, experimental context, consistency with independent findings, and separation of demonstrated results from proposed interpretation.

### 2.2. Genetic Evidence

The principal genetic foundation is the Fu-185, identified at a false-discovery rate (FDR) of ≤0.05 in the rare-coding-variant study of Fu et al. [24]. This analysis extended the Satterstrom-102, identified at FDR ≤ 0.10 [23]. These statistically defined sets provide a broad but bounded starting point assembled without selection for the proposed S2M mechanism. Membership is treated as evidence of association with ASD ascertainment, not specificity to ASD, sensory manifestations, or S2M; many included genes also contribute to intellectual disability, developmental delay, epilepsy, and other neurodevelopmental phenotypes [23,24].

The Fu-185 was selected because it arose from large human datasets rather than pathway nomination, integrated several classes and sources of rare coding variation, and was sufficiently extensive to test whether an independently assembled ASD gene set contained support for the prespecified mechanism [24]. Placement within S2M was nevertheless restricted. Genes lacking a sufficiently specific mechanistic position remained outside the counted hierarchy regardless of the strength of their ASD association.

Complementary evidence was drawn from additional exome, whole-genome, copy-number, and inherited-variant studies [22,25,26,42] and, where informative, SFARI Gene [43]. These sources were not treated as statistically independent replications because studies may share participants, contributing cohorts, analytical methods, or earlier evidence, and SFARI Gene is a curated synthesis rather than an independent experiment. Complementary evidence permits inclusion of experimentally informative genes whose ASD-genetic support differs from ASC-core membership, but those categories remain visibly distinct in the text, tables, and figures.

### 2.3. Criteria for Mechanistic Placement

Genes were evaluated individually rather than assigned solely through ontology terms, pathway databases, coexpression networks, protein-interaction maps, or broad functional labels. Placement required experimental evidence connecting the encoded product to a defined operation within the proposed directional sequence.

1. **Generation and organization of the activity-dependent Ca^2+^ signal.** Level 1 includes proteins regulating membrane excitability, neurotransmitter-receptor signaling, Ca^2+^ entry, phosphoinositide metabolism, receptor-associated scaffolding, IP_3_R-mediated release, clearance, or spatial organization [28,29,30,31,32,33]. Direct physical interaction with an IP_3_R is not required; a channel or receptor qualifies when it determines the electrical, synaptic, biochemical, or spatial conditions from which the Ca^2+^ signal is generated.
2. **Ca^2+^-dependent decoding.** Level 2 includes proteins that respond to the magnitude, duration, frequency, localization, or repetition of Ca^2+^ activity and translate that information into electrical, synaptic, metabolic, biochemical, or transcriptional consequences [5,7,8,9,10,34,35,36]. Ca^2+^ responsiveness alone is insufficient without evidence that the protein interprets or transmits information carried by the signal.
3. **Transcriptional and chromatin memory.** Level 3 includes proteins that help establish, maintain, constrain, or terminate activity-induced transcriptional and chromatin states [5,8,9,10,11,36]. Here, *memory* denotes a persistent but reversible cellular change that influences subsequent responsiveness, not behavioral or cognitive memory. General involvement in chromatin regulation or neurodevelopment is insufficient without evidence connecting the protein to activity-responsive transcription or stabilization of its consequences.

Several proteins span level boundaries and were assigned according to their best-supported principal operation, with cross-level functions stated explicitly. Modified future responsiveness is treated as the output of the three levels rather than as a fourth molecular level. Evidence of habituation, homeostatic plasticity, sensory adaptation, or another form of experience-dependent recalibration can strengthen a placement but cannot substitute for evidence identifying the molecular operation involved [1,2,3,4,5,6,7,36].

Protein interaction, transcriptional response, pathway annotation, or coexpression alone was not sufficient. A physical interaction may be context restricted and may not establish directional function; a transcriptionally Ca^2+^-responsive gene need not decode Ca^2+^; and a chromatin regulator may control developmental specification without stabilizing an activity-induced program. Evidence from non-neural systems was considered when the biochemical relationship was well established, but the limitation of the cellular context was retained.

Three forms of mechanistic evidence were distinguished. **Direct Ca^2+^-dependent evidence** indicates that a protein binds Ca^2+^ or Ca^2+^–calmodulin, functions as a Ca^2+^ sensor, or displays experimentally demonstrated Ca^2+^-dependent activity. **Ca^2+^-pathway-mediated evidence** indicates regulation through a defined intermediary downstream of Ca^2+^ without direct Ca^2+^ sensing by the assigned protein. **Activity-dependent evidence** indicates regulation following neuronal stimulation without establishing Ca^2+^ as the sole or immediate mediator. These forms were not treated as equivalent, and broader activity dependence did not establish direct regulation by IP_3_R-generated Ca^2+^.

The principal safeguard was therefore:

*Association with ASD, neuronal activity, Ca*^2+^ *responsiveness, transcription, or chromatin regulation alone is insufficient to place a gene within the proposed directional hierarchy.*

### 2.4. Mechanistic-Evidence Categories

Genes were assigned one principal mechanistic designation. **Core** mechanistic components directly perform a defined operation within one of the three levels. Experimentally supported mechanistic **Bridges** connect successive operations or levels through a demonstrated directional relationship. **Candidate** components or modifiers have genetic and functional relevance but lack sufficient evidence for placement as direct components of the sequence. **Outside** the presently mapped axis denotes ASD-associated genes for which no sufficiently specific position can currently be established; it does not imply weak genetic evidence or biological irrelevance.

These categories describe the status and directness of mechanistic placement, not the strength of association with ASD. Genetic evidence was therefore recorded independently, including ASC-core membership [23,24], complementary published findings [22,25,26,37,42], variant-level evidence, and curated status [43]. This two-dimensional system prevents strongly associated genes from being assigned without an adequate mechanism while permitting essential molecular components to be represented without overstating their genetic support. *ITPR1* and *ITPR3*, for example, occupy the same core Level 1 channel operation but carry different genetic evidence; *PPP3CA*/calcineurin occupies a well-established Level 2 decoding position while its ASD-genetic support remains complementary.

The resulting classification should therefore be read as an evidence-graded mechanistic synthesis rather than as a new gene-discovery or enrichment analysis. It is intentionally conservative and may underestimate the contribution of the proposed axis. Its purpose is to distinguish strongly supported placements, provisional components, and genes that presently remain outside the map (Box 1).

Box 1 **Genetic association and mechanistic placement are independent dimensions.**

 **Genetic support** asks whether variation in a gene is associated with ASD and how that association was established. **Mechanistic placement** asks whether the experimentally established function of its product connects it specifically to Ca^2+^-signal construction, decoding, or stabilization of an activity-induced state. Strong evidence in one dimension does not confer strong evidence in the other.

 The IP_3_R family illustrates the distinction. *ITPR1* and *ITPR3* are counted as core Level 1 genes, whereas *ITPR2* is discussed as a functional family modifier. *ITPR3* belongs to the Fu-185; *ITPR1* belongs to neither the Fu-185 nor the Satterstrom-102 but carries complementary ASD and neurodevelopmental evidence; and *ITPR2* remains genetically less resolved. Equivalent mechanistic positions therefore should not be mistaken for equivalent genetic support.

## 3. From Signal to Memory: The Adaptive Hierarchy

Neuronal activity is transient, but adaptation requires selected consequences to outlast the initiating signal. The proposed S2M framework organizes this transformation into three successive functional tasks: generation and organization of the activity-dependent Ca^2+^ signal, interpretation of that signal, and stabilization of selected consequences as persistent but reversible changes in cellular state [7,8,9,10,11,27,28,29,30,31,32,33,34,35,36]. These positions are analytically distinguishable but biologically interdependent. They do not constitute a single invariant cascade, and different cells may use different molecular components to perform the same general operations.

### 3.1. Level 1: Signal Generation and Organization

Level 1 converts synaptic and membrane activity into a dynamically organized intracellular Ca^2+^ signal. Voltage-gated channels determine whether and how neurons fire; excitatory and inhibitory receptors define the synaptic input; NMDA receptors and voltage-gated Ca^2+^ channels provide routes for Ca^2+^ entry; metabotropic receptors engage phospholipase C and phosphoinositide signaling; and molecular scaffolds organize these components within membrane, synaptic, and ER domains [27,28,29,30,31,32,33]. Pumps, exchangers, store filling, organellar contacts, and metabolic state further shape signal duration, localization, and recovery.

IP_3_Rs occupy a distinctive but nonexclusive convergence position. Receptor-generated IP_3_ activates ER Ca^2+^ release, while IP_3_R gating integrates IP_3_ with cytosolic and luminal Ca^2+^, nucleotides, phosphorylation, interacting proteins, and feedback [27,28,30,31]. Ca^2+^ entry and intracellular release interact, and the resulting signals differ in amplitude, duration, frequency, oscillatory structure, localization, and propagation [29,31,34,35]. Level 1 lesions are therefore predicted to alter the structure of the signal presented to downstream machinery rather than necessarily producing a uniform increase or decrease in Ca^2+^.

### 3.2. Level 2: Ca^2+^-Dependent Decoding

A Ca^2+^ signal has no fixed biological meaning outside the machinery that interprets it. Level 2 contains electrical, biochemical, synaptic, metabolic, and transcriptional decoders that discriminate among signal patterns. Calmodulin, CaMKII, calcineurin, and Ca^2+^-activated ion channels provide direct or closely coupled decoding, whereas CREB, MEF2, and associated regulators receive Ca^2+^-pathway-mediated or broader activity-dependent input [8,9,10,33,34,35,44,45,46]. Decoder output depends on signal dynamics, subcellular localization, developmental state, and the molecular complement of the responding cell.

The distinction between signal generation and interpretation is experimentally important. A properly generated signal may be misinterpreted when a Ca^2+^-responsive channel, kinase, phosphatase, or transcriptional regulator is disrupted; intact decoders cannot reconstruct temporal or spatial information already lost at Level 1. Level 2 lesions are therefore predicted to produce a mismatch between Ca^2+^ dynamics and their immediate cellular consequences.

### 3.3. Level 3: Transcriptional and Chromatin Memory

Level 3 connects interpreted activity to a more durable regulatory substrate. Activity-responsive transcription factors recruit coactivators, corepressors, histone-modifying enzymes, nucleosome remodelers, and other chromatin-associated complexes to selected loci [8,9,10,11]. These processes can determine whether a transcriptional response remains transient, is reinforced through repeated activation, or becomes stabilized as a longer-lasting cellular program.

The resulting defect need not be a simple loss of transcription. A normally adaptive program may be insufficiently stabilized, persist inappropriately, or engage the wrong loci. Placement at Level 3 therefore requires evidence connecting a chromatin regulator to activity-responsive transcription or stabilization of its consequences; general roles in chromatin regulation, neuronal differentiation, or ASD association are insufficient.

### 3.4. Adaptive Output and Testable Predictions

The hierarchy culminates in a changed cell rather than in transcription alone. Persistent changes in receptors, channels, synaptic proteins, cytoskeletal organization, metabolic capacity, organelle structure, transcription, or chromatin accessibility can alter how the cell responds when stimulation recurs [5,7,8,9,10,11,32,36,47]. These downstream changes feed back upon signal generation and decoding, making the organization directional during an adaptive cycle but recurrent across repeated experience:

*Activity → Ca* ^2+^ *-signal generation → decoding → transcriptional and chromatin memory → modified responsiveness → altered processing of subsequent activity*

The proposed framework predicts distinct earliest abnormalities. A Level 1 lesion should disrupt membrane activity or Ca^2+^-signal organization. A Level 2 lesion should preserve at least part of the initiating signal but produce an inappropriate electrical, biochemical, synaptic, metabolic, or transcriptional response. A Level 3 lesion should permit initial signaling and interpretation but impair the persistence, termination, or genomic specificity of the resulting state. These different defects could nevertheless converge on an abnormal response trajectory across repeated stimulation.

The common predicted phenotype is therefore not a uniform baseline abnormality but a failure of prior activity to modify later responsiveness appropriately. Adaptation may be insufficient, excessive, delayed, unstable, overly persistent, or generalized beyond the stimulus that elicited it. Testing S2M requires dynamic experiments that measure initial stimulation, repeated exposure, recovery, renewed challenge, and responsiveness to novelty. The framework would be strengthened if the earliest abnormal step corresponded to a gene’s assigned position and variant-specific correction restored the response trajectory. It would require revision if the predicted ordering or convergence were not observed.

The following sections apply these criteria to the genes assigned to each level, beginning with the electrical, synaptic, biochemical, and spatial mechanisms that generate and organize activity-dependent Ca^2+^ signals.

## 4. Level 1: Generation and Organization of the Activity-Dependent Ca^2+^ Signal

Level 1 contains the molecular operations that determine the Ca^2+^ signal presented to the rest of the cell. These include membrane excitability, synaptic transmission, Ca^2+^ entry, phosphoinositide signaling, IP_3_R-mediated endoplasmic-reticulum (ER) Ca^2+^ release, spatial organization, and recovery between stimuli. Placement at this level does not require direct physical interaction with an IP_3_R; it requires evidence that the gene product controls the activity, molecular coupling, or cellular geometry from which the intracellular Ca^2+^ signal is constructed.

### 4.1. Electrical Initiation and Control

***SCN2A.*** *SCN2A* belongs to the Fu-185 and encodes Na_v_1.2, a voltage-gated Na^+^ channel expressed predominantly in excitatory neurons [23,24,48]. Loss- and gain-of-function variants can produce different physiological and clinical consequences, but the relevant Level 1 operation is consistent: Na_v_1.2 regulates action-potential initiation and dendritic electrogenesis and thereby determines which synaptic events recruit voltage-gated Ca^2+^ entry and downstream intracellular signaling [48,49].

***SCN1A.*** *SCN1A* also belongs to the Fu-185 and encodes Na_v_1.1, a voltage-gated Na^+^ channel with a major role in inhibitory interneurons [23,24,50,51]. Altered Na_v_1.1 function changes inhibitory control and therefore the frequency, synchrony, and duration of network activity presented to Ca^2+^-entry and intracellular-release mechanisms. ASD-associated missense variants identified by Fu et al. include changes in the Ca^2+^–calmodulin-regulated carboxy-terminal region, further linking membrane excitability to Ca^2+^-dependent feedback [24,52].

***KCNQ3.*** *KCNQ3* belongs to the Fu-185 and encodes K_v_7.3, a component of neuronal M-current channels that restrain subthreshold excitability and repetitive firing [23,24,53]. Functionally characterized ASD-associated variants specifically alter S4 voltage-sensor residues that increase channel activity, shifting activation toward more hyperpolarized potentials and reducing neuronal responsiveness [54]. *KCNQ3* therefore demonstrates that either increased or decreased channel function can distort the electrical activity from which downstream Ca^2+^ signals are generated.

These channels converge physiologically rather than through stable physical interaction. *SCN2A* regulates excitatory-neuron firing, *SCN1A* strongly influences interneuron and circuit activity, and *KCNQ3* provides a voltage-dependent brake on repetitive firing. Each can alter the timing, intensity, and cellular distribution of Ca^2+^ entry, transmitter release, receptor activation, and intracellular Ca^2+^ mobilization.

### 4.2. Excitatory and Inhibitory Synaptic Input

***GRIN2B* and *GRIA2.*** *GRIN2B* and *GRIA2* belong to the Fu-185 [24]. *GRIN2B* encodes the GluN2B NMDA-receptor subunit, linking glutamatergic transmission directly to Ca^2+^ entry and to signaling complexes that couple synaptic activity to plasticity and transcription [55,56,57]. *GRIA2* encodes the GluA2 AMPA-receptor subunit, whose RNA editing and subunit composition strongly influence AMPA-receptor Ca^2+^ permeability [58]. Their Level 1 placement reflects control of excitatory drive and the amount, timing, and route of activity-dependent Ca^2+^ entry.

***GABRB3* and *GABBR2.*** *GABRB3* and *GABBR2* also belong to the Fu-185 [24]. *GABRB3* encodes a GABA_a_-receptor subunit that regulates fast inhibitory conductance, whereas *GABBR2* encodes an essential GABA_β_-receptor subunit that engages G proteins to regulate ion channels and second-messenger pathways [59,60,61]. Neither receptor is an IP_3_R component. Their Level 1 relevance lies in determining the inhibitory and metabotropic context in which neurons generate Ca^2+^ signals.

Together, these receptors respond to the major excitatory and inhibitory neurotransmitters—glutamate and GABA, respectively—thereby determining how excitation and inhibition are transformed into membrane voltage, Ca^2+^ entry, transmitter release, and engagement of intracellular signaling. Their placement does not imply that all variants move the system in the same direction; it identifies the synaptic operation through which each lesion first enters the proposed hierarchy.

### 4.3. Ca^2+^ Entry and Channel Organization

***CACNA1A.*** *CACNA1A* is not a Fu-185 or Satterstrom-102 gene and is retained through complementary ASD/NDD and mechanistic evidence. It encodes the pore-forming Ca_v_2.1 subunit, a principal mediator of presynaptic Ca^2+^ entry and neurotransmitter release [62,63]. Its Level 1 placement reflects direct control of the Ca^2+^ influx that couples action potentials to vesicle fusion and shapes downstream intracellular signaling.

***CACNA1E.*** *CACNA1E* belongs to the Fu-185 and encodes the Ca_v_2.3 channel [24,64]. Ca_v_2.3 contributes to neuronal Ca^2+^ entry, excitability, and transmitter release. It therefore occupies Level 1 as a direct determinant of the amplitude and localization of activity-dependent Ca^2+^ influx.

***CACNA2D3.*** *CACNA2D3* is included in the Satterstrom-102 but not the Fu-185 [23,24]. It encodes an α_2_δ auxiliary subunit that regulates trafficking and functional expression of voltage-gated Ca^2+^ channels [65,66]. Its placement is consequently organizational: it modifies where and how effectively pore-forming Ca^2+^ channels operate rather than forming the conducting pore itself.

The three genes illustrate distinct ways of controlling Ca^2+^ entry: pore formation, channel subtype-specific physiology, and auxiliary-subunit-dependent trafficking. These distinctions are retained even though all three occupy the same broad Level 1 operation.

### 4.4. Spatial Scaffolds and Signaling Domains

***HOMER1.*** *HOMER1* belongs to neither the Fu-185 nor the Satterstrom-102 and is retained through complementary genetic and mechanistic evidence [23,24,67,68,69,70]. Homer proteins organize metabotropic glutamate receptors, Shank scaffolds, and IP_3_Rs within postsynaptic signaling domains. *HOMER1* is therefore placed at Level 1 as a spatial bridge that can regulate receptor-to-PLC–IP_3_–IP_3_R coupling without itself generating IP_3_ or conducting Ca^2+^.

***SHANK2 and SHANK3.*** *SHANK2* and *SHANK3* belong to the Fu-185 [24,71,72,73]. They encode postsynaptic scaffolds that organize glutamate receptors, Homer proteins, cytoskeletal elements, and signaling complexes. Their Level 1 placement reflects the spatial assembly of synaptic components that convert receptor activation into appropriately localized electrical and intracellular signals; it does not imply that all consequences of SHANK disruption are mediated by IP_3_Rs.

***ANK2.*** *ANK2* belongs to both the Satterstrom-102 and Fu-185 [23,24]. Ankyrin-B organizes membrane proteins, cytoskeletal structures, and intracellular membrane domains and has been implicated in neuronal excitability, axonal organization, and organelle positioning [74]. It is retained as a Level 1 spatial organizer whose disruption could alter the geometry through which membrane activity is coupled to intracellular Ca^2+^ signaling.

These scaffolds emphasize that signal construction depends on proximity as well as molecular abundance. A receptor, enzyme, and Ca^2+^ channel may all remain present while weakened spatial coupling reduces the efficiency, localization, or reproducibility of the resulting Ca^2+^ signal.

### 4.5. Phosphoinositide Signaling

***PLCB1.*** *PLCB1* does not belong to the ASC-core set and is retained through complementary ASD/NDD and mechanistic evidence [23,24,43,75,76]. PLCβ1 hydrolyzes phosphatidylinositol 4,5-bisphosphate to generate IP_3_ and diacylglycerol downstream of G-protein-coupled receptors. It therefore occupies a direct Level 1 position between receptor activation and IP_3_R-mediated ER Ca^2+^ release.

***INPP1.*** *INPP1* also does not belong to the ASC-core set and is retained through complementary genetic and mechanistic evidence [23,24,77,78,79]. It encodes a metal-dependent, Li^+^-inhibited phosphomonoesterase involved in inositol-phosphate metabolism [80]. By contributing to inositol-phosphate turnover and the substrate economy of phosphoinositide signaling, INPP1 can influence pathway recycling and recovery between successive episodes of signaling [79,80,81]. It participates in inositol-phosphate metabolism and phosphoinositide recycling. Its placement is less direct than that of *PLCB1*: INPP1 can influence the substrate economy and recovery of repeated phosphoinositide signaling rather than catalyzing the immediate production of IP_3_.

The distinction is important. *PLCB1* contributes to initiation of an IP_3_ signal, whereas *INPP1* may influence how effectively the pathway can be reset and re-engaged. Both can therefore alter repeated activity, but through different positions in the signaling cycle.

### 4.6. IP_3_ Receptors as an Intracellular Convergence Point

IP_3_Rs are tetrameric ER Ca^2+^-release channels regulated by IP_3_, cytosolic and luminal Ca^2+^, ATP, phosphorylation, and interacting proteins [28,30,31,82]. Their integrative gating allows electrical, synaptic, and metabotropic inputs to be converted into local, oscillatory, or propagating Ca^2+^ signals. IP_3_Rs are therefore an important convergence mechanism within Level 1, not the exclusive source or universal causal center of neuronal Ca^2+^ signaling.

***ITPR1.*** *ITPR1* belongs to neither the Fu-185 nor the Satterstrom-102 and is retained through complementary ASD-variant, neurodevelopmental, and mechanistic evidence [23,24,26,37,38]. It encodes IP_3_R1, the predominant neuronal IP_3_R isoform. Pathogenic alleles cause a spectrum of neurological disorders, including Gillespie syndrome and spinocerebellar ataxias, and certain non-null Gillespie-associated alleles can act through dominant-negative effects in multimeric channels [37]. These findings establish the sensitivity of neuronal function to altered IP_3_R1 channel assembly and gating without implying that all *ITPR1*-related disease is mediated through the proposed sensory-homeostasis mechanism.

***ITPR3.*** *ITPR3* belongs to the Fu-185 and encodes IP_3_R3 [24]. The BTBR *T+ Itpr3*^tf^/J mouse provides unusually informative experimental evidence for its Level 1 placement. The *Itpr3*^tf^ allele contains a 12-base-pair in-frame deletion that removes residues 983–986; low-abundance altered protein remains detectable, distinguishing the allele from a protein null [39,83]. BTBR mice show markedly reduced responses to sweet, umami, bitter, Polycose, and calcium stimuli. These inputs use distinct upstream receptors but converge substantially on PLCβ2-dependent IP_3_ production, IP_3_R3-mediated Ca^2+^ release, and TRPM5-dependent depolarization in taste-receptor cells [39,40,41]. Mapping, congenic replacement, targeted-null comparison, and failure of targeted *Itpr3*-null alleles to complement the recessive *tufted* hair phenotype establish that *Itpr3*^tf^ causes the taste and hair-patterning phenotypes [39,40,83]. Contribution of the allele to the broader behavioral phenotypes of the BTBR strain remains untested.

IP_3_R1, IP_3_R2, and IP_3_R3 subunits can assemble into homo- and heterotetrameric channels [28,84]. Gillespie-associated *ITPR1* alleles provide precedent that a non-null altered IP_3_R subunit can disrupt a multimeric channel [37]. Residual altered IP_3_R3 in the *tf* mouse could likewise affect homo- or heterotetrameric assemblies, potentially producing effects different from complete loss of IP_3_R3—a testable hypothesis.

Together, human ASD-genetic association through the Fu-185 and the BTBR experiments support *ITPR3* at Level 1. *ITPR1* occupies the same core channel position but carries a distinct, complementary genetic evidence grade. *ITPR2* remains a functionally relevant family modifier rather than a counted core gene because its ASD-genetic support and specific contribution are less resolved.

### 4.7. Recovery, Repeated Activity, and Metabolic Coupling

***ATP2B2.*** *ATP2B2* belongs to the Fu-185 and encodes PMCA2, a high-affinity plasma-membrane Ca^2+^ pump enriched in neurons and sensory cells [24,85]. By extruding Ca^2+^ after stimulation, PMCA2 regulates signal duration and recovery. It is therefore classified as a Level 1 regulator of termination rather than as a component of the PLC–IP_3_–IP_3_R sequence.

***DNM1* and *SYNJ1.*** Neither *DNM1* nor *SYNJ1* belongs to the Fu-185 or Satterstrom-102 [23,24]. DNM1 mediates membrane fission during activity-dependent vesicle retrieval, whereas SYNJ1 remodels phosphoinositides during vesicle uncoating and recycling [86,87]. *SYNJ1* has complementary ASD-genetic support from postzygotic-mosaic analysis [88], and pathogenic variants in both genes cause developmental and epileptic encephalopathies [89,90]. They are retained as modifiers of the repeated synaptic activity presented to Level 1, not as core PLC–IP_3_–IP_3_R components.

***KCNMA1.*** *KCNMA1* belongs to both the Satterstrom-102 and Fu-185 and encodes the BK Ca^2+^- and voltage-activated K^+^ channel [23,24,91]. Because BK channels translate local Ca^2+^ elevation into rapid repolarizing feedback, *KCNMA1* lies at the Level 1–Level 2 boundary. It is classified principally as an electrical decoder in Level 2 but is shown here because its feedback immediately reshapes subsequent excitability and Ca^2+^ entry.

***ER–mitochondrial coupling.*** IP_3_Rs at ER–mitochondrial contacts can generate local Ca^2+^ microdomains that support mitochondrial Ca^2+^ uptake and oxidative metabolism [32,47]. None of the canonical VDAC–MCU uptake genes or principal tethering and organelle-dynamics genes examined here belong to the Fu-185 or Satterstrom-102. ER–mitochondrial coupling is therefore retained as a mechanistically important downstream extension rather than as an independently reconstructed ASC-core module.

### 4.8. Predicted Consequence of Level 1 Disruption

**Figure 1:**
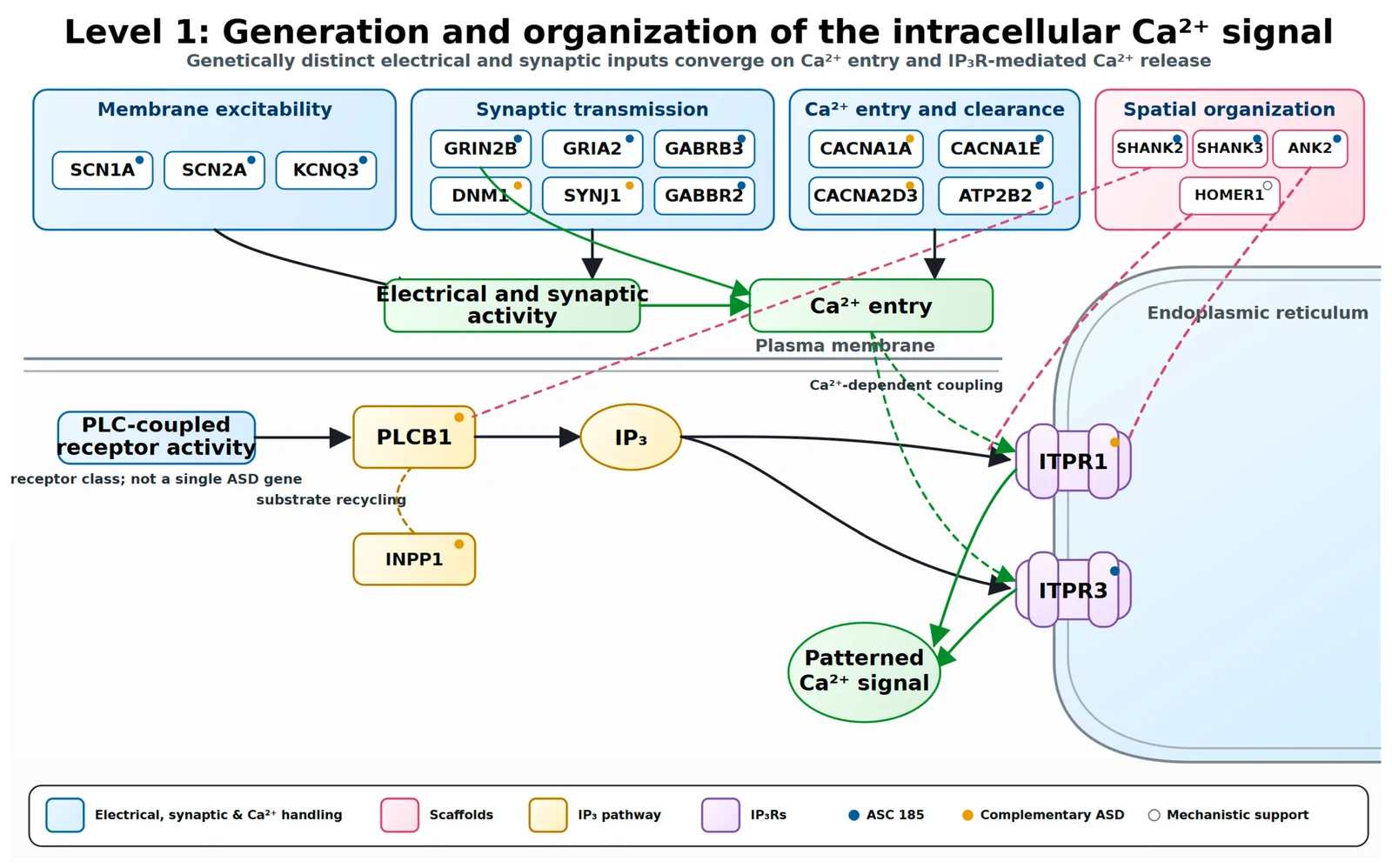
Level 1: Generation and organization of the intracellular Ca^2+^ signal.

A Level 1 lesion is predicted to alter the Ca^2+^ signal presented to the cell—its amplitude, duration, frequency, localization, propagation, or recovery. The direction need not be uniform: different variants may increase, decrease, mistime, or spatially redistribute activity. The defining experiment is therefore simultaneous measurement of the initiating electrical or receptor event and the resulting Ca^2+^ dynamics across initial and repeated stimulation. A Level 1 defect should be detectable in signal construction before abnormalities in downstream decoding or chromatin persistence are invoked (Table 1 and Figure 1).

Genetically distinct ion channels, neurotransmitter receptors, synaptic proteins, signaling enzymes, pumps, and scaffolds regulate membrane excitability, synaptic transmission, Ca^2+^ entry, PLC-dependent IP_3_ production, IP_3_R-mediated ER Ca^2+^ release, and recovery between stimuli. *SCN2A*, *SCN1A*, and *KCNQ3* regulate membrane activity; *GRIN2B*, *GRIA2*, *GABRB3*, and *GABBR2* shape synaptic input; *CACNA1A*, *CACNA1E*, and *CACNA2D3* regulate Ca^2+^ entry; and *ATP2B2* contributes to Ca^2+^ clearance. *DNM1* and *SYNJ1* shape repeated synaptic activity. *PLCB1* generates IP_3_, whereas *INPP1* supports phosphoinositide recycling. *HOMER1*, *SHANK2*, *SHANK3*, and *ANK2* organize receptor-, scaffold-, cytoskeletal-, and ER-associated signaling domains. IP_3_ activates channels assembled from IP_3_R1, IP_3_R3, and potentially other IP_3_R isoform subunits, generating patterned intracellular Ca^2+^ signals. Black solid arrows indicate principal signal flow; green solid arrows indicate Ca^2+^ entry, release, or contribution to the patterned Ca^2+^ signal; green dashed lines indicate Ca^2+^-dependent coupling; pink dashed lines indicate spatial organization; and the gold dashed line indicates substrate recycling. Box color encodes functional category; blue, gold, and open-gray dots denote Fu-185 membership, complementary ASD/NDD evidence, and mechanistic support only, respectively. *ITPR3* belongs to the Fu-185, whereas *ITPR1* carries complementary ASD/NDD support and belongs to neither the Fu-185 nor the Satterstrom-102 [23,24,37,38].

## 5. Level 2: Decoding the Activity-Dependent Ca^2+^ Signal

Level 2 contains proteins that interpret features of the Ca^2+^ signal and convert them into electrical, synaptic, biochemical, or transcriptional responses. Direct Ca^2+^ sensors, Ca^2+^–calmodulin-regulated enzymes, and defined downstream transcriptional routes provide the strongest placements. Broader activity dependence is informative but is not treated as equivalent to direct Ca^2+^ dependence.

### 5.1. Decoding Principles

Ca^2+^ signals carry information through amplitude, duration, frequency, localization, and repetition [34,35,36]. Calmodulin and other sensors translate these dynamics into changes in kinase, phosphatase, channel, and transcription-factor activity. The same Ca^2+^ transient can therefore produce different outcomes depending on which decoders are present, where they are located, and how rapidly they activate and recover [44,45,46].

### 5.2. CAMK2A: Phosphorylation-Dependent Decoding

*CAMK2A* belongs to the Fu-185 and encodes the α subunit of Ca^2+^/calmodulin-dependent protein kinase II [24]. Binding of Ca^2+^–calmodulin relieves autoinhibition, and intersubunit phosphorylation at Thr286 can prolong activity after Ca^2+^ declines [92,93,94]. Repeated Ca^2+^ pulses can therefore be integrated according to their frequency and spacing, giving CaMKII a direct mechanistic basis for Level 2 placement [95,96,97,98,99].

CaMKIIα regulates synaptic receptors, ion channels, cytoskeletal proteins, and transcriptional pathways [92,93,94,100,101]. Its placement does not imply that every *CAMK2A* phenotype results from IP_3_R-generated Ca^2+^. It identifies a well-established decoder through which temporally structured Ca^2+^ signals can be converted into phosphorylation-dependent changes that outlast the initiating transient.

### 5.3. PPP3CA/Calcineurin: Dephosphorylation-Dependent Decoding

*PPP3CA* is not a Fu-185 or Satterstrom-102 gene and is retained through complementary ASD/NDD and mechanistic evidence [23,24,102,103,104]. It encodes calcineurin Aα, the catalytic component of a Ca^2+^/calmodulin-dependent phosphatase. Ca^2+^ binding to calcineurin B and calmodulin relieves autoinhibition and permits substrate dephosphorylation [46].

Calcineurin is not merely a reversal mechanism for CaMKII. Differences in Ca^2+^ sensitivity, kinetics, localization, and substrates allow the kinase and phosphatase to extract complementary information from the same signal. *PPP3CA* is therefore placed as a core biochemical Level 2 decoder whose genetic support is reported separately from its strong mechanistic evidence.

### 5.4. HDAC4–MEF2C and YWHAG: Activity-Dependent Transcriptional Derepression

*HDAC4* and *MEF2C* belong to the Fu-185 [24]. *YWHAG*, which encodes 14-3-3γ, also belongs to the Fu-185 and provides a genetic anchor for the phospho-dependent trafficking machinery [24]. Although HDAC4 belongs to the class IIa histone deacetylase family, its intrinsic catalytic activity toward conventional acetyl-lysine substrates is extremely weak [105]. HDAC4 therefore functions predominantly as a mobile transcriptional corepressor scaffold rather than as a conventional histone deacetylase.

Ca^2+^-responsive kinase pathways phosphorylate HDAC4, promoting 14-3-3 binding, nuclear export or cytoplasmic retention, and relief of HDAC4-mediated repression of MEF2-dependent transcription [8,9,10,100,106,107,108,109].

MEF2C supplies DNA-binding and target specificity, whereas HDAC4 functions predominantly as a mobile corepressor scaffold rather than as a strong conventional lysine deacetylase [101,106]. Calcineurin provides a complementary route by enhancing neuronal MEF2 DNA binding and transcription through dephosphorylation-dependent mechanisms [107,110]. The module can therefore integrate kinase-dependent corepressor removal with phosphatase-dependent modification of MEF2 itself.

Pathogenic disruption of *HDAC4* and *MEF2C* causes distinct neurodevelopmental syndromes [111,112]. These observations support the importance of the module but do not establish that every clinical consequence arises from impaired Ca^2+^-dependent transcription. The module is placed at the Level 2–Level 3 boundary: it interprets activity through regulated trafficking and dephosphorylation while controlling access to a longer-lasting transcriptional program.

### 5.5. CAMTA2 and the CAMTA Family

*CAMTA2* belongs to the Fu-185, whereas *CAMTA1* belongs to neither ASC-core set [23,24]. Both encode calmodulin-binding transcription activators with conserved DNA-binding, ankyrin-repeat, IQ, and calmodulin-binding domains. This architecture makes CAMTA proteins plausible Ca^2+^–calmodulin-linked transcriptional decoders, but responses to defined neuronal Ca^2+^ waveforms have not been established.

The best-characterized mammalian *CAMTA2* mechanism is signal-responsive transcription in cardiomyocytes involving class IIa HDACs [113]. Stronger neuronal evidence concerns *CAMTA1*: reduction in adult mouse hippocampus impairs long-term contextual and spatial memory, and work in invertebrate neurons with a single CAMTA locus links the family to calmodulin expression and Ca^2+^ signaling [114,115]. A homozygous regulatory *CAMTA2* variant has been implicated in syndromic tremulous dystonia, whereas pathogenic *CAMTA1* variants cause a predominantly cerebellar neurological disorder [116,117].

The branch is therefore retained through complementary evidence: *CAMTA2* supplies the Fu-185 genetic anchor and a demonstrated signal-responsive transcriptional mechanism outside neurons, while *CAMTA1* supplies stronger neuronal family-level evidence. Direct demonstration of *CAMTA2*-dependent Ca^2+^ decoding in mammalian neurons remains necessary.

### 5.6. CREB–CREBBP/EP300: Transcriptional Decoding

CREB1 is not a Fu-185 or Satterstrom-102 gene and is retained for mechanistic support; *CREBBP* belongs to the Fu-185, whereas *EP300* belongs to neither the ASC-core set but has complementary NDD evidence [23,24,38]. Activity-dependent kinase pathways phosphorylate CREB, including at Ser133, enabling recruitment of CREBBP/CBP and related coactivators [8,9,10,118,119,120,121]. Nuclear Ca^2+^ and CaMKIV contribute to this route, while calcineurin-dependent dephosphorylation and nuclear entry of CREB-regulated transcription coactivators provide a complementary Ca^2+^- and cAMP-sensitive input [118,119,120].

CREBBP is a multidomain coactivator and lysine acetyltransferase. Its recruitment links immediate transcriptional decoding to chromatin regulation by assembling transcriptional machinery and acetylating histones and regulatory proteins [118,119,121,122,123,124]. Activity-dependent CREB encounters at CREBBP-dependent acetylation sites further support a coupled rather than strictly sequential relationship between transcription-factor activation and chromatin competence [122].

*This module spans Levels 2 and 3. CREB and its upstream kinases and phosphatases interpret the Ca*^2+^ *signal; CREBBP and EP300 help determine whether that interpretation becomes a productive and potentially persistent transcriptional state. Their placement does not imply that CREBBP or EP300 acts exclusively downstream of Ca*^2+^ *signaling.*

### 5.7. KCNMA1 and Immediate Electrical Decoding

Introduced in Section 4 as a boundary component, *KCNMA1* belongs to both ASC-core sets and provides an immediate electrical form of Ca^2+^ decoding [23,24,91]. BK channels combine membrane depolarization with local Ca^2+^ elevation and convert those inputs into K^+^ efflux, altered action-potential waveform, firing, and transmitter release [91,125]. Their position near Ca^2+^ sources allows them to decode brief local nanodomain signals on a millisecond timescale.

Both gain- and loss-of-function *KCNMA1* variants cause neurological disease, and their effects depend on cellular context [126,127]. A de novo rearrangement disrupting *KCNMA1* in an individual with autism and intellectual disability produced haploinsufficiency and reduced BK-channel activity [128]. *KCNMA1* is therefore classified as a genetically supported electrical decoder at the Level 1–Level 2 boundary: it does not carry Ca^2+^ information directly to chromatin, but it rapidly changes the activity and Ca^2+^ signals presented to slower decoders.

### 5.8. Predicted Consequence of Level 2 Disruption

A Level 2 lesion is predicted to preserve at least part of the initiating Ca^2+^ signal while producing an inappropriate electrical, biochemical, synaptic, or transcriptional response. The defect may involve sensitivity, timing, localization, or persistence rather than a simple increase or decrease in output. The strongest test therefore measures the Ca^2+^ signal and decoder response together. A relatively intact Ca^2+^ transient accompanied by abnormal CaMKII activation, calcineurin signaling, BK current, transcription-factor trafficking, or activity-dependent gene expression would support a decoding defect.

**Figure 2:**
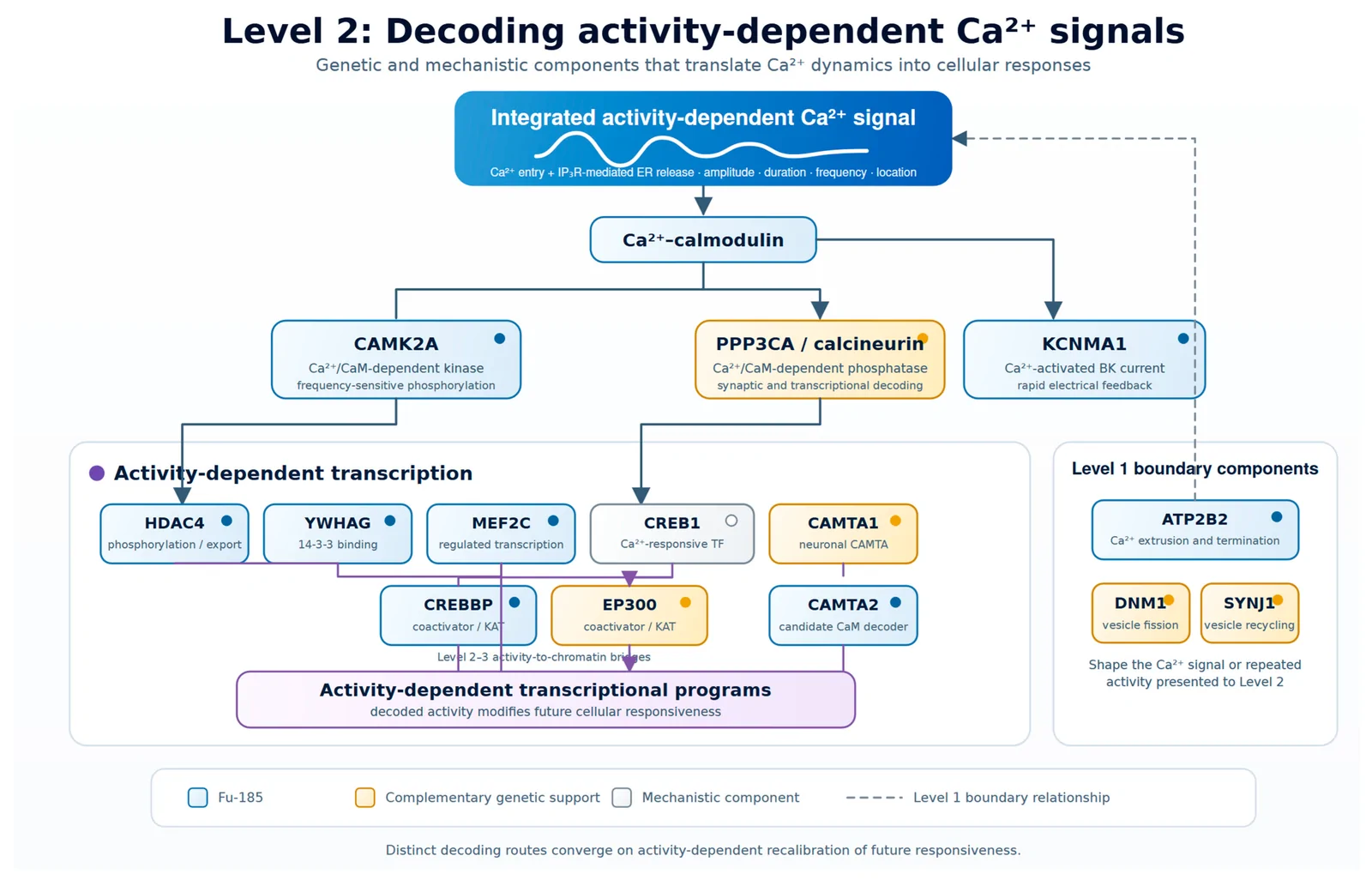
Level 2: Decoding activity-dependent Ca^2+^ signals.

Level 2 marks the transition from construction of an activity-dependent Ca^2+^ signal to its biological interpretation (Table 2 and Figure 2). Whether selected consequences acquire appropriate persistence depends on the transcriptional and chromatin-regulatory mechanisms considered in Section 6.

Ca^2+^–calmodulin activates kinase and phosphatase decoders that translate the magnitude, timing, repetition, and location of activity-dependent Ca^2+^ signals into electrical, synaptic, and transcriptional responses. CAMK2A and PPP3CA/calcineurin provide complementary phosphorylation- and dephosphorylation-dependent decoding mechanisms [92,93,94,102,103,104]. HDAC4–14-3-3 trafficking—genetically represented here by *HDAC4* and *YWHAG*—connects phosphorylation-dependent signal decoding to regulation of MEF2C-dependent transcription [100,101,106,107]. CAMTA2 provides a candidate route from calmodulin signaling to transcription, whereas CAMTA1 is retained as a mechanistically informative neuronal family member. *CAMTA2* supplies the Fu-185 genetic anchor for this branch; the more direct neuronal and memory-related evidence concerns CAMTA1 [24,113,114,115]. The CREB–CREBBP/EP300 pathway provides an additional route from decoded activity to transcription, with CREBBP and EP300 extending across the Level 2–Level 3 boundary through transcriptional coactivation and lysine acetylation [118,119,120,121]. KCNMA1 converts local Ca^2+^ elevations directly into rapid electrical feedback [91]. ATP2B2, DNM1, and SYNJ1 are Level 1 boundary components that regulate Ca^2+^-signal termination or the repeated synaptic activity presented to the decoding machinery [85,86,87]. These distinct routes are proposed to converge on activity-dependent modification of future cellular responsiveness. Blue, gold, and open-gray styling denote Fu-185 membership, complementary ASD/NDD genetic support, and mechanistic-component status, respectively. The dashed gray line indicates a Level 1 boundary relationship.

## 6. Level 3: Transcriptional and Chromatin Memory

Level 3 contains mechanisms that help establish, maintain, constrain, or terminate transcriptional and chromatin states initiated by neuronal activity. Here, *memory* denotes a persistent but reversible activity-dependent change in cellular state that influences subsequent responsiveness, not behavioral or cognitive memory. Strong ASD association alone is insufficient for placement: the mechanistic evidence must connect the gene product to activity-responsive transcription or to stabilization of its consequences.

### 6.1. From Transcriptional Response to Altered Cellular State

Immediate transcription does not by itself establish cellular memory. A response can remain transient unless transcription-factor occupancy, coactivator recruitment, histone modification, nucleosome organization, and other regulatory processes alter the probability that relevant loci will be engaged again. Level 3 therefore addresses the genomic persistence and specificity of an interpreted signal, not simply the amount of transcription produced during stimulation.

### 6.2. CREBBP and EP300: Coactivation and Acetylation

*CREBBP* belongs to the Fu-185 and encodes CREB-binding protein, a transcriptional coactivator and lysine acetyltransferase [24]. Its Ca^2+^-regulated recruitment was considered in Section 5 [119,122]. At Level 3, the relevant operation is the assembly of transcriptional machinery and acetylation of histones and regulatory proteins, which can support enhancer and promoter competence at selected activity-responsive loci [118,123,124]. CREBBP therefore provides a genetically anchored bridge between immediate transcriptional decoding and chromatin-supported cellular memory.

*EP300* belongs to neither the Fu-185 nor the Satterstrom-102 and is retained through complementary NDD and mechanistic evidence [23,24,38]. It encodes p300, a close but noninterchangeable paralogue of CBP with overlapping coactivator and lysine-acetyltransferase functions [118]. CREBBP and EP300 are consequently placed in the same functional module but retain different genetic evidence grades.

### 6.3. KDM6B: Removal of Repressive Chromatin Marks

*KDM6B* belongs to both the Satterstrom-102 and Fu-185 and encodes a histone demethylase that removes H3K27me2/3, including the repressive H3K27me3 mark [23,24,129]. Neuronal activity induces *Kdm6b* in hippocampal neurons, and experimental reduction impairs induction of a subset of activity-regulated genes and activity-dependent neuronal survival [130]. Pathogenic variants cause a neurodevelopmental disorder that frequently includes ASD or autistic features [131].

KDM6B is therefore placed as a Level 3 chromatin bridge: it can lower a Polycomb-associated barrier that would otherwise limit expression of selected activity-responsive programs. This placement does not imply that all KDM6B functions are Ca^2+^-dependent or that every developmental consequence reflects defective adaptive transcription.

### 6.4. HDAC4–MEF2C Across the Level 2–Level 3 Boundary

*HDAC4* and *MEF2C* belong to the Fu-185 [24]. Section 5 described their role in interpreting activity through phosphorylation-dependent HDAC4 trafficking and calcineurin-dependent regulation of MEF2 [100,101,106,107]. At Level 3, the relevant operation is reversible control of MEF2-bound loci: nuclear HDAC4 maintains repression, whereas its export permits MEF2-dependent transcription and associated chromatin changes.

Pathogenic disruption of *HDAC4* and *MEF2C* causes neurodevelopmental abnormalities [132,133]. The module is therefore a genetically supported bridge rather than a simple switch. Its output depends on neuronal identity, developmental stage, cofactors, chromatin context, and activity pattern, and it can either permit or constrain selected transcriptional programs.

### 6.5. CHD2: Candidate Activity-Responsive Nucleosome Remodeling

*CHD2* belongs to both the Satterstrom-102 and Fu-185 families and encodes an ATP-dependent chromatin remodeler [23,24]. CHD2 haploinsufficiency alters CHD2 occupancy, H3K27 acetylation, developmental gene expression, and differentiation of human cortical interneuron progenitors [134]. These findings establish a role in neuronal chromatin competence and development.

The directional connection to Ca^2+^ signaling is less direct than for CREBBP or HDAC4–MEF2C. CHD2 has not been shown to be recruited by a canonical Ca^2+^-responsive transcription factor or to remodel chromatin specifically downstream of IP_3_R-mediated Ca^2+^ release. It is therefore classified as a genetically supported candidate Level 3 stabilizer. Demonstration of activity-dependent CHD2 recruitment at Ca^2+^-responsive loci would strengthen its placement as a direct bridge.

### 6.6. Broader ASD-Associated Chromatin Machinery

The broader Level 3 set contains four functional modules, all genetically anchored but more variably connected to activity-responsive transcription [23,24,135,136,137,138]. Histone-state regulators include *ASH1L*, *EHMT1*, *KMT5B*, and *SETD5*, together with the replication-independent histone H3.3 gene *H3F3A/H3-3A*. Nucleosome-remodeling genes include *CHD8* and *ARID1B*. *POGZ* and *ADNP* contribute to chromatin architecture and developmental transcriptional regulation, whereas *DNMT3A* establishes longer-lasting DNA-methylation states.

These genes provide strong evidence that chromatin regulation contributes to ASD, but their inclusion does not establish that each protein is a direct target of Ca^2+^ signaling or a component of a single biochemical cascade. Most are therefore treated as candidate downstream executors or stabilizers whose disruption may alter the genomic context in which activity-dependent transcription occurs. Stronger placement would require evidence of activity-dependent recruitment or modification, action at Ca^2+^-responsive loci, or control of transcriptional persistence after stimulation.

### 6.7. Predicted Consequence of Level 3 Disruption

A Level 3 lesion is predicted to permit initial signal generation and at least partial decoding while disrupting the persistence, termination, or genomic specificity of the resulting transcriptional state. An appropriate program may decay too rapidly, persist inappropriately, or engage the wrong loci. CREBBP dysfunction could weaken activity-linked acetylation, KDM6B disruption could preserve repressive H3K27 methylation, HDAC4–MEF2C abnormalities could alter derepression, and CHD2 dysfunction could impair nucleosome reorganization [100,101,106,107,118,119,120,121,122,123,129,130,131,132,133,134].

Testing this prediction requires measurements beyond the initial response. Ca^2+^ dynamics and immediate decoder activation should be measured first, followed over time by transcription-factor occupancy, chromatin accessibility, histone modifications, and gene expression during repeated stimulation, recovery, and renewed challenge. The defining pattern would be a relatively preserved initiating signal and immediate response accompanied by abnormal persistence, termination, reinstatement, or stimulus specificity of the regulatory program.

**Figure 3:**
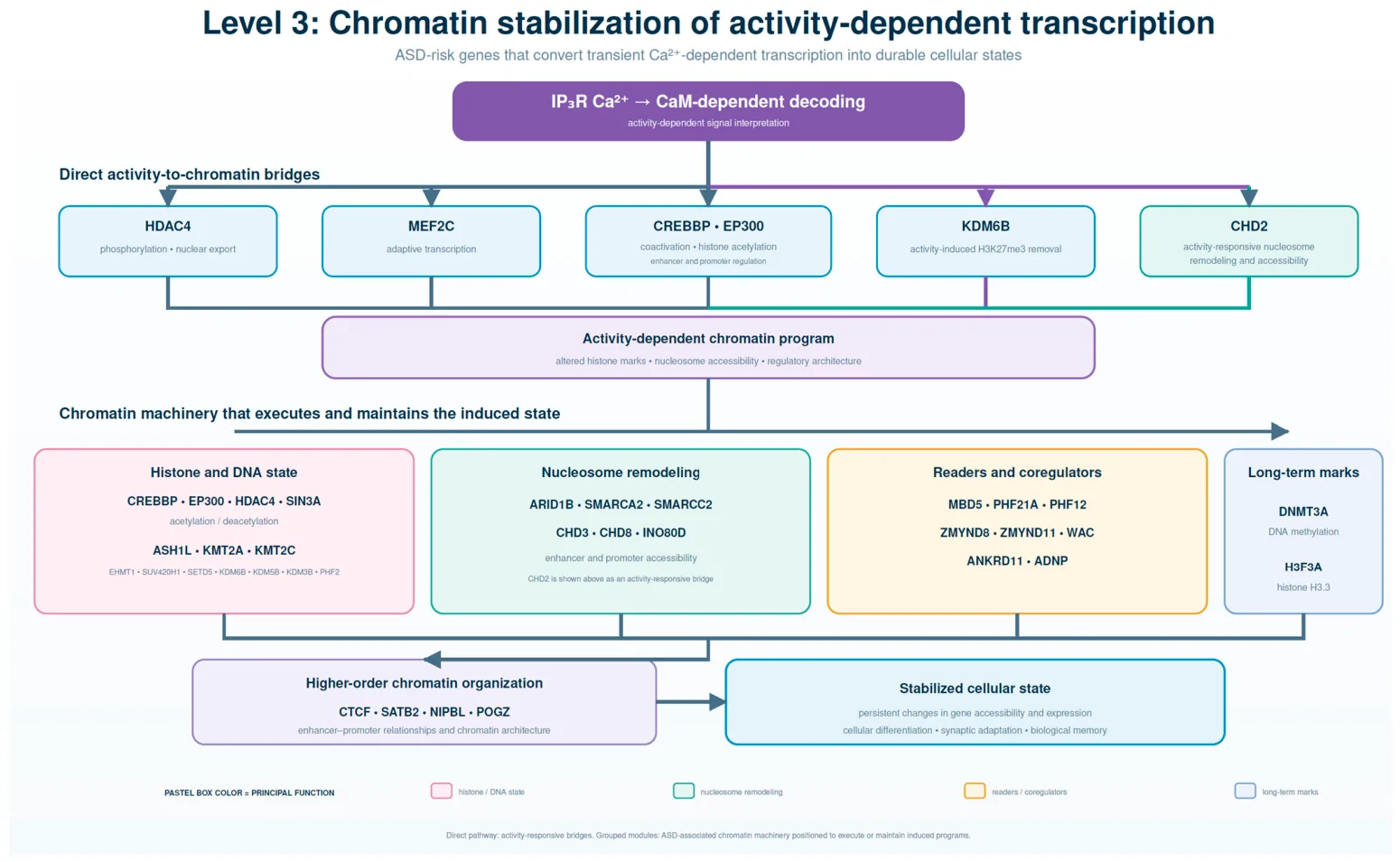
Chromatin stabilization of activity-dependent transcription.

Level 3 is deliberately narrower than the full collection of ASD-associated chromatin regulators. It identifies a defensible intersection between ASD genetics and the mechanisms that could convert transient activity-dependent transcription into a persistent but reversible cellular trace. It does not imply equivalent genetic or mechanistic evidence across genes, nor does it establish that impaired sensory homeostasis mediates every associated phenotype (Table 3 and Figure 3).

Level 3 begins with activity-dependent transcriptional output arising downstream of Ca^2+^ signals, including IP_3_R-mediated ER Ca^2+^ release interpreted by Ca^2+^/calmodulin-dependent decoders at Level 2. HDAC4–MEF2C, CREBBP/EP300, and KDM6B provide the most direct activity-to-chromatin bridges developed in the text, whereas CHD2 provides a candidate connection to nucleosome remodeling and chromatin accessibility. HDAC4 nuclear export relieves repression of MEF2-dependent transcription; CREBBP and EP300 couple activated transcription factors to lysine acetylation and enhancer–promoter regulation; and KDM6B removes repressive H3K27me3. These mechanisms converge on an activity-dependent chromatin program that may be executed, constrained, or maintained by broader ASD-associated machinery regulating histone and DNA state, nucleosome organization, transcriptional coregulation, long-term chromatin marks, and higher-order chromatin organization. The grouped genes are positioned as candidate downstream executors or stabilizers and are not all established direct targets of IP_3_R-mediated Ca^2+^ signaling. Together, these processes may help convert transient activity-dependent transcription into persistent but reversible changes in gene accessibility, expression, synaptic adaptation, and cellular state. Pastel box colors denote principal Level 3 functional categories rather than evidence strength; blue, gold, and open-gray dots denote Fu-185 membership, complementary ASD/NDD evidence, and mechanistic support only, respectively.

## 7. Integration of the Three-Level Adaptive Hierarchy

### 7.1. From Signal Generation to Adaptive Cellular Memory

**Figure 4:**
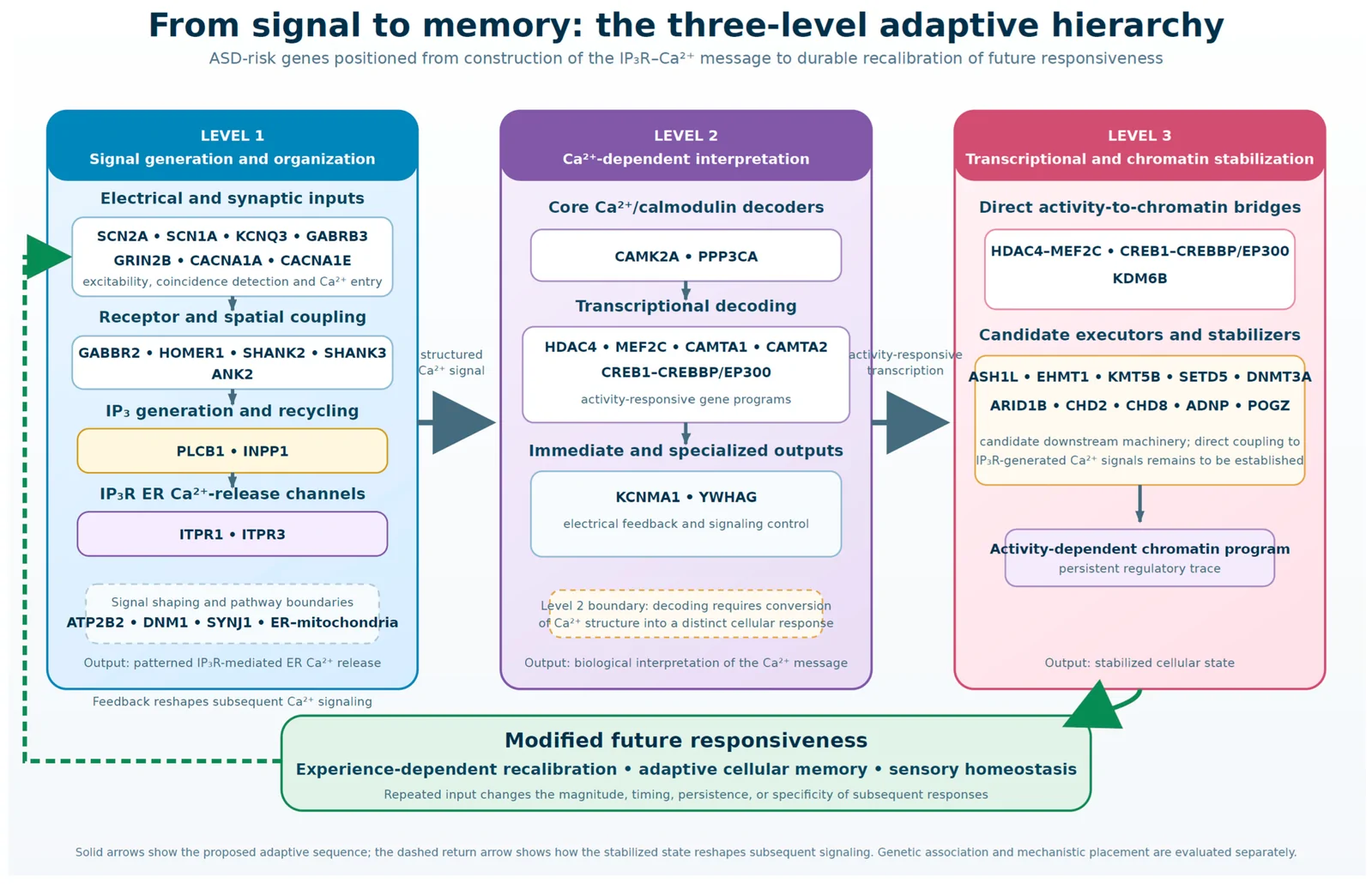
From signal to memory: the proposed three-level adaptive hierarchy.

The three levels developed in Section 4, Section 5 and Section 6 represent successive functional tasks through which neuronal activity could modify future cellular responsiveness. Level 1 constructs and spatially organizes intracellular Ca^2+^ signals. Level 2 interprets those signals through electrical, biochemical, synaptic, metabolic, and transcriptional decoders. Level 3 determines whether selected transcriptional consequences acquire—or relinquish—the persistence and genomic specificity needed to influence later cellular behavior. Together, these levels define the proposed progression from signal generation and organization through biological interpretation to adaptive cellular memory (Table 4 and Figure 4).

The hierarchy is directional but neither linear nor exclusive. Activity-dependent transcription can alter receptors, ion channels, signaling proteins, Ca^2+^ pumps, decoders, metabolic enzymes, and synaptic components [8,9,10]. These changes can subsequently modify excitability, synaptic strength, organelle organization, and metabolic capacity, making the endpoint of one adaptive cycle part of the initial condition for the next. Adaptive cellular memory therefore denotes a persistent but reversible change in how a cell processes subsequent information; it does not imply behavioral or cognitive memory.

Lesions entering at different levels are predicted to produce distinguishable proximal abnormalities. A Level 1 lesion should alter construction or organization of the Ca^2+^ signal. A Level 2 lesion should permit at least partial signal generation but disrupt its electrical, biochemical, synaptic, metabolic, or transcriptional interpretation. A Level 3 lesion should permit initial signaling and interpretation while impairing the persistence, termination, or genomic specificity of the resulting regulatory program. These distinct defects could nevertheless converge on an abnormal response trajectory across repeated stimulation even when resting Ca^2+^, basal transcription, or the initial response appears relatively normal.

Sensory homeostasis provides a physiologically coherent context in which to test this prediction. Repeated sensory input must generate an appropriately structured intracellular signal, recruit a proportionate interpretation, and leave a sufficiently durable but reversible cellular trace to influence the next encounter. Failure at any stage could prevent predictable stimuli from losing salience, suppress responses to biologically important change, or contribute to unstable shifts among hyperresponsiveness, hyporesponsiveness, and sensory seeking. These remain testable hypotheses connecting cellular response dynamics to sensory phenotype rather than established consequences of disruption of the mapped genes.

The levels should therefore be understood as assigned principal mechanistic positions rather than rigid gene classes. HDAC4–MEF2C and CREB–CREBBP cross the boundary between decoding and chromatin regulation, whereas ATP2B2, KCNMA1, DNM1, and SYNJ1 influence more than one stage through feedback on Ca^2+^ dynamics, excitability, or repeated synaptic transmission. IP_3_Rs likewise represent one important convergence mechanism within a broader Ca^2+^-signaling network that includes NMDA receptors, voltage-gated Ca^2+^ channels, ryanodine receptors, store-operated entry, mitochondrial Ca^2+^ handling, and plasma-membrane extrusion. Such overlap reflects biological continuity rather than failure of the framework.

Level 1 constructs and organizes activity-dependent Ca^2+^ signals through electrical, synaptic, phosphoinositide, spatial-organizing, and IP_3_R-mediated mechanisms. Level 2 decoders translate the resulting Ca^2+^ dynamics into electrical, biochemical, synaptic, and transcriptional responses. Level 3 activity-to-chromatin bridges and candidate downstream regulators are positioned to establish or stabilize selected transcriptional consequences as persistent but reversible cellular states. These states may modify future responsiveness by altering the molecular conditions under which subsequent signals are generated and interpreted. Solid arrows show the proposed progression from signal construction through decoding and transcriptional or chromatin regulation to modified future responsiveness. The dashed return arrow represents the proposed feedback through which the resulting cellular state reshapes subsequent Level 1 signaling. Box and column colors distinguish functional positions within the hierarchy; they do not indicate the strength of ASD-genetic or mechanistic evidence, which is reported separately in Table 1, Table 2, Table 3 and Table 4.

### 7.2. Representation of the Fu-185 Within S2M

The consolidated S2M hierarchy contains 44 distinct genes. After gene-symbol normalization, 32 belong to the prespecified Fu-185 [24]. The hierarchy therefore contains 32/185 genes (17.3%) from that cohort, while 32/44 genes (72.7%) in the mapped hierarchy carry direct support from the primary Fu analysis.

The 32 Fu-185 genes are distributed across all three levels: 13 in Level 1, 6 in Level 2, and 13 in Level 3. Thus, 19/32 (59.4%) occupy Levels 1 and 2, where they contribute to the construction or interpretation of activity-dependent Ca^2+^ signals. The remaining 13 occupy Level 3, connecting these signaling consequences to transcriptional and chromatin regulation. Genetic representation therefore spans the full proposed sequence, with a prominent concentration in Ca^2+^-signal construction and interpretation.

The IP_3_R-family assignments illustrate why genetic status and mechanistic position must remain separate. *ITPR3* belongs to the Fu-185. *ITPR1* carries complementary ASD-variant and neurodevelopmental evidence but belongs to neither the Fu-185 nor the Satterstrom-102. *ITPR2* is discussed as a functionally relevant family modifier but is not included among the 44 mapped core genes because its ASD-genetic support and specific contribution remain insufficiently resolved.

These values describe representation rather than statistically independent enrichment. ASD-genetic evidence contributed to construction of the hierarchy, making an overlap test against the same source cohort circular. Formal enrichment would require a pathway defined independently of the tested cohort, a prespecified genomic background, fixed inclusion rules, and consistent handling of paralogues, complexes, aliases, and boundary components. Gene Ontology, KEGG, and related analyses could provide complementary descriptions of the Fu-185 but would address a different question and would not establish the directional relationships proposed here. The generalizability of the proposed hierarchy is instead evaluated in a companion analysis using independently derived genetic, protein-interaction, and cross-disorder datasets.

## 8. Limitations

S2M is a hypothesis-driven narrative review and structured mechanistic synthesis rather than a systematic review, gene-discovery analysis, unsupervised enrichment study, or definitive classification of ASD-associated genes. The Fu-185 was identified independently of the proposed framework, but assigning individual genes to Levels 1–3 required interpretation of genetic, biochemical, cellular, and neurophysiological evidence. Explicit criteria and separate grading of genetic and mechanistic support improve transparency but do not eliminate judgment. S2M should therefore be regarded as a bounded and testable working map of one biologically coherent subset of ASD genetics.

The starting gene set also imposes boundaries. The Fu-185 arose principally from rare coding-variant association and does not capture the full contribution of common, noncoding, structural, regulatory, or highly polygenic variation [24]. Membership indicates association with ASD ascertainment rather than specificity to ASD; many included genes also contribute to intellectual disability, developmental delay, epilepsy, or other neurodevelopmental phenotypes. Moreover, primary and complementary genetic sources may share participants, cohorts, methods, or earlier discovery samples, while curated resources such as SFARI Gene synthesize rather than independently replicate published evidence. The reported 44-gene and 32-gene counts therefore quantify representation, not independent enrichment.

Genes within the hierarchy occupy unequal evidentiary positions. Some combine strong ASD association with a well-defined mechanistic placement; others have compelling mechanistic relevance but weaker or differently classified genetic support; still others are retained as bridges, boundary components, or candidate modifiers. The IP_3_R family exemplifies this distinction: *ITPR3* belongs to the Fu-185, *ITPR1* carries complementary ASD and neurodevelopmental evidence, and *ITPR2* remains outside the counted core. Related molecular functions do not imply equivalent genetic support.

Mechanistic evidence is similarly uneven. Voltage-gated channels, CAMK2A, calcineurin, and CREBBP/EP300 have extensively characterized functions, whereas other placements depend on particular cell types, developmental stages, species, or non-neuronal systems. Several Level 2 or Level 3 proteins have not been shown to respond directly to an IP_3_R-generated Ca^2+^ waveform. Direct Ca^2+^ dependence, regulation through a defined Ca^2+^-responsive intermediary, and broader activity dependence were therefore kept distinct. Placement identifies the operation through which a protein could influence signal interpretation or persistence; it does not establish one uninterrupted biochemical pathway downstream of IP_3_R activation.

The three levels simplify a recurrent, branched, and cell-type-dependent system. Several proteins act at more than one position, and the same protein may serve different functions across neuronal populations or developmental periods. Level assignments indicate the principal position at which a lesion is predicted to enter the adaptive sequence; they do not define isolated compartments or an invariant order of events. Later compensation may also obscure the earliest molecular abnormality.

Variant heterogeneity creates an additional limitation. Variants in the same gene may cause loss or gain of function, altered gating, impaired trafficking, mislocalization, abnormal protein interactions, or developmental changes in expression. These effects can produce different and sometimes opposing physiological consequences. Gene-level placement therefore predicts the general operation most likely to be disturbed, not the direction, magnitude, timing, or cell-type specificity of every variant. The predictions ultimately require variant-specific correction and recreation in relevant human cellular and neuronal systems.

IP_3_Rs are proposed as an important convergence mechanism, not the exclusive source of neuronal Ca^2+^ signals or a universal causal center of ASD. NMDA receptors, voltage-gated Ca^2+^ channels, ryanodine receptors, store-operated entry, mitochondrial handling, and plasma-membrane extrusion all contribute to neuronal Ca^2+^ dynamics. The emphasis on IP_3_Rs reflects their capacity to integrate receptor-generated IP_3_ with Ca^2+^, energy state, phosphorylation, and interacting proteins, together with the genetic and experimental evidence developed in Section 4, Section 5 and Section 6.

The BTBR *T+ Itpr3*^tf^/J model provides evidence that multiple independent sensory inputs can converge on a shared IP_3_R-mediated intracellular Ca^2+^ release mechanism, as mapping, congenic replacement, targeted-null comparison, and failure of targeted *Itpr3*-null alleles to complement the recessive *tufted* hair phenotype establish that *Itpr3*^tf^ causes the multiple taste and hair-patterning phenotypes [39,40,83]. However, the allele’s contribution to the broader behavioral and somatic phenotypes of the BTBR strain remains untested.

Finally, *sensory homeostasis* is a proposed physiological output, not an established consequence of the mapped sequence. Sensory phenotypes may arise through peripheral, synaptic, circuit, attentional, predictive, or behavioral mechanisms not represented in S2M. Cellular adaptation cannot be assumed to equal perceptual habituation, and peripheral-cell abnormalities cannot be assumed to reproduce neuronal or circuit-level processing. Most available studies also emphasize baseline measurements or single stimuli and therefore cannot demonstrate impaired adaptive cellular memory. Testing S2M requires repeated stimulation, recovery, renewed challenge, and a novel comparison stimulus with sufficient temporal resolution to locate the earliest abnormal step. Until those experiments are performed, S2M remains a falsifiable framework rather than a causal explanation of ASD or its sensory manifestations.

## 9. Conclusions and Future Directions

Large-scale ASD sequencing has identified genes distributed across membrane excitability, synaptic transmission, intracellular signaling, transcription, and chromatin regulation. The S2M analysis shows that these apparently disparate categories can be organized into a directional biological framework centered on how prior neuronal activity modifies future cellular responsiveness. The consolidated hierarchy contains 44 genes, 32 of which belong to the independently defined Fu-185. It therefore captures 17.3% of that primary ASD gene set, while 72.7% of the genes placed within the hierarchy carry direct Fu-185 support. This degree of representation argues that activity-dependent signal construction, interpretation, and stabilization constitute a prominent axis of ASD genetic vulnerability rather than an isolated pathway assembled from a few selected examples.

The distribution of the 32 Fu-185 genes strengthens this interpretation. Thirteen occupy Level 1, six occupy Level 2, and thirteen occupy Level 3. Thus, 19/32 (59.4%) contribute to the construction or interpretation of activity-dependent Ca^2+^ signals, while the remainder connect those signaling consequences to transcriptional and chromatin regulation. ASD-associated variation is therefore represented across the full progression from membrane and synaptic activity to persistent cellular change. IP_3_Rs provide one potentially important point of intracellular convergence within this broader network, but the framework does not require every lesion to act through the same channel, Ca^2+^ source, or molecular complex.

The principal implication for ASD is that genetic heterogeneity may converge on a shared physiological problem without producing a uniform baseline abnormality. A channel variant, signaling-enzyme defect, transcriptional-decoder lesion, and chromatin-regulatory mutation may disturb different molecular events, yet each could weaken the relationship between prior activity and subsequent responsiveness. The relevant common phenotype may therefore be an abnormal response trajectory across repeated stimulation—insufficient, excessive, mistimed, unstable, or inappropriately generalized adaptation—rather than a single direction of change in resting Ca^2+^, excitability, gene expression, or sensory responsiveness.

This prediction creates a practical diagnostic strategy. Functional assays should move beyond static measurements and single-stimulus responses to quantify signal generation, decoding, transcriptional engagement, persistence, recovery, and response to renewed or novel stimulation. Such challenge-based trajectories could reveal abnormalities that are invisible at baseline, distinguish lesions entering at different levels, and provide functional evidence for variants whose consequences remain uncertain. Peripheral cells, induced pluripotent-stem-cell-derived neurons, organoids, and genetically defined animal models may each contribute, provided that their relationship to neuronal and sensory phenotypes is established directly.

The hierarchy also suggests a more precise therapeutic logic. Treatment should target the earliest abnormal operation rather than a distal phenotype produced after multiple compensatory and developmental changes. This approach distinguishes interventions aimed at signal construction, decoding, or stabilization and aligns therapy with the affected developmental stage.

A Level 1 defect may require correction of channel function, receptor coupling, Ca^2+^ entry, intracellular release, localization, or clearance. A Level 2 defect may require restoration of electrical, enzymatic, or transcriptional decoding. A Level 3 defect may require normalization of the persistence or genomic specificity of an activity-induced program. Because gain- and loss-of-function variants in the same gene can produce opposing physiological effects, intervention must be both variant-specific and level-aware. The goal is not simply to increase or suppress signaling, but to restore an appropriate relationship between stimulation, cellular response, and adaptation.

Sensory homeostasis provides an experimentally accessible and clinically relevant setting in which to test this model. Failure of activity-dependent recalibration could contribute to sensory hyperresponsiveness, hyporesponsiveness, or sensory seeking without requiring these manifestations to share an identical proximal cause. Demonstrating that connection will require coordinated measurements of molecular response trajectories, neuronal and circuit adaptation, and quantitative sensory phenotypes before and after variant correction or targeted intervention.

The decisive test of S2M is therefore prospective. For a genetically defined lesion, the framework should predict the earliest abnormal step, the downstream trajectory across repeated stimulation, and the intervention capable of restoring that trajectory. Success would provide a mechanistic bridge from ASD-associated variation to cellular physiology, offer dynamic biomarkers for stratification and treatment monitoring, and identify level- and variant-specific therapeutic targets. Failure of these predictions would require revision or rejection of the assigned relationships. In this form, S2M is not merely a classification of ASD-associated genes; it is a falsifiable strategy for converting genetic heterogeneity into experimentally distinguishable mechanisms and, ultimately, more precise diagnostic and therapeutic approaches (Box 2).

Box 2 **The decisive experimental test: measure adaptation, not only response.**

 The defining prediction concerns the trajectory of responsiveness across time rather than a baseline measurement or the response to one stimulus.

 **Experimental sequence**

 *Initial stimulus → repeated stimulation → recovery interval → renewed familiar stimulus → novel comparison stimulus*

 At each stage, measurements should span the hierarchy:

- **Level 1:** membrane activity, synaptic input, Ca^2+^ entry, and the amplitude, duration, frequency, and spatial organization of intracellular Ca^2+^ release.
- **Level 2:** CAMK2A and calcineurin activation, Ca^2+^-dependent electrical feedback, transcription-factor activation, and coupling between the Ca^2+^ waveform and its immediate consequences.
- **Level 3:** transcription, chromatin accessibility, histone state, regulatory persistence, and stimulus specificity.
- **Functional output:** magnitude, timing, persistence, recovery, and specificity of the response to subsequent stimulation.

 **Discriminating predictions**

- A Level 1 lesion should produce an abnormal Ca^2+^ signal from the initial response.
- A Level 2 lesion should preserve at least part of the initiating signal but produce a mismatch between that signal and its electrical, synaptic, biochemical, or transcriptional consequences.
- A Level 3 lesion should permit initial signal generation and interpretation but impair the persistence, specificity, or later effect of the induced regulatory state.
- Successful variant-specific rescue should restore the response trajectory, not merely normalize one baseline value.

 The framework would be supported if these distinct proximal abnormalities converge on impaired recalibration during repeated stimulation. It would require revision if the predicted ordering is not observed or if lesions assigned to different levels do not converge on a measurable abnormality of future responsiveness.

## Figures and Tables

**Table 1:** Level 1 generation and organization of the intracellular Ca^2+^ signal. Signal-generating, organizing, phosphoinositide, scaffold, and IP_3_R components.

Gene	Evidence Category	Functional Category	Principal S2M Function	Mechanistic Placement
*ATP2B2*	Fu-185	Electrical, synaptic, and Ca^2+^ handling	Extrudes cytosolic Ca^2+^ and controls the amplitude and termination of Ca^2+^ signals	Ca^2+^-homeostatic modifier
*CACNA1A*	Complementary ASD/NDD	Electrical, synaptic, and Ca^2+^ handling	Mediates presynaptic Ca^2+^ entry and neurotransmitter release through CaV2.1 channels	Core signal-generating component
*CACNA1E*	Fu-185	Electrical, synaptic, and Ca^2+^ handling	Mediates activity-dependent Ca^2+^ entry through CaV2.3 channels	Core signal-generating component
*CACNA2D3*	Complementary ASD/NDD	Electrical, synaptic, and Ca^2+^ handling	Regulates trafficking and function of voltage-gated Ca^2+^ channels	Signal-organizing component
*DNM1*	Complementary ASD/NDD	Electrical, synaptic, and Ca^2+^ handling	Couples neuronal activity to synaptic-vesicle endocytosis and recycling	Activity-dependent synaptic component
*GABBR2*	Fu-185	Electrical, synaptic, and Ca^2+^ handling	Regulates GABA type B receptor control of neuronal excitability and voltage-gated Ca^2+^ channels	Upstream synaptic organizer
*GABRB3*	Fu-185	Electrical, synaptic, and Ca^2+^ handling	Regulates fast GABAergic transmission, developmental excitability, and recruitment of Ca^2+^ entry	Upstream electrical organizer
*GRIA2*	Fu-185	Electrical, synaptic, and Ca^2+^ handling	Controls AMPA-receptor conductance and Ca^2+^ permeability	Core signal-generating component
*GRIN2B*	Fu-185	Electrical, synaptic, and Ca^2+^ handling	Provides NMDA-receptor coincidence detection and activity-dependent Ca^2+^ entry	Core signal-generating component
*KCNQ3*	Fu-185	Electrical, synaptic, and Ca^2+^ handling	Controls subthreshold excitability through M-current; ASD-associated missense variants alter positively charged residues in the S4 voltage-sensor helix	Core signal-generating component
*SCN1A*	Fu-185	Electrical, synaptic, and Ca^2+^ handling	Regulates inhibitory-neuron firing; its C-terminal region supports Ca^2+^–calmodulin-dependent channel regulation	Core signal-generating component
*SCN2A*	Fu-185	Electrical, synaptic, and Ca^2+^ handling	Generates action potentials and controls depolarization-dependent Ca^2+^ recruitment	Core signal-generating component
*SYNJ1*	Complementary ASD/NDD	Electrical, synaptic, and Ca^2+^ handling	Couples phosphoinositide metabolism to synaptic-vesicle recycling	Synaptic/phosphoinositide bridge
*ANK2*	Fu-185	Scaffold and spatial organization	Organizes membrane–cytoskeletal and ER-associated signaling domains, including IP_3_R localization	Core signal-organizing scaffold
*HOMER1*	Complementary ASD/NDD	Scaffold and spatial organization	Couples metabotropic glutamate receptors, Shank scaffolds, and IP_3_Rs	Experimentally supported mechanistic bridge
*SHANK2*	Fu-185	Scaffold and spatial organization	Organizes postsynaptic receptor and signaling complexes	Core signal-organizing scaffold
*SHANK3*	Fu-185	Scaffold and spatial organization	Organizes postsynaptic receptor, Homer, and signaling complexes	Core signal-organizing scaffold
*INPP1*	Complementary ASD/NDD	IP_3_ pathway	Regulates phosphoinositide recycling and substrate availability for IP_3_ generation	Candidate pathway modifier
*PLCB1*	Complementary ASD/NDD	IP_3_ pathway	Generates IP_3_ and diacylglycerol downstream of receptor activation	Core biochemical pathway component
*ITPR1*	Complementary ASD/NDD	IP_3_R channel	Forms an IP_3_-gated ER Ca^2+^-release channel at the proposed intracellular convergence point	Core mechanistic component
*ITPR3*	Fu-185	IP_3_R channel	Forms an IP_3_-gated ER Ca^2+^-release channel and mediates convergence of receptor-distinct sensory inputs	Core mechanistic component

Evidence categories: Fu-185, membership in the primary ASC-derived 185-gene set; complementary ASD/NDD, other published autism or neurodevelopmental-disorder genetic evidence; mechanistic support only, functional or interaction evidence without qualifying genetic support. Functional groupings are identified in words and separated by horizontal rules. Fu-185 membership records genetic association and does not by itself establish a direct signal-to-memory mechanism.

**Table 2:** Level 2 decoding of intracellular Ca^2+^ signals. Electrical, biochemical, trafficking, and transcriptional decoders that assign biological meaning to Ca^2+^ dynamics.

Gene	Evidence Category	Functional Category	Principal S2M Function	Mechanistic Placement
*CAMK2A*	Fu-185	Ca^2+^/calmodulin decoding	Decodes Ca^2+^–calmodulin dynamics through phosphorylation and activity-dependent molecular persistence	Core Ca^2+^ decoder
*PPP3CA*	Complementary ASD/NDD	Ca^2+^/calmodulin decoding	Decodes Ca^2+^–calmodulin through calcineurin-dependent dephosphorylation, synaptic remodeling, and transcription	Core Ca^2+^ decoder
*KCNMA1*	Fu-185	Electrical output	Converts local Ca^2+^ and depolarization into BK-channel opening and altered excitability	Immediate electrical Ca^2+^ decoder
*HDAC4*	Fu-185	Protein trafficking	Couples Ca^2+^-regulated nucleocytoplasmic trafficking to MEF2-dependent transcriptional repression	Experimentally supported Level 2–3 bridge
*YWHAG*	Fu-185	Protein trafficking	Regulates phosphorylation-dependent trafficking and localization of signaling proteins	Signal-decoding modifier
*CAMTA1*	Complementary ASD/NDD	Transcriptional decoding	Supports neuronal CAMTA-family functions in activity-dependent transcription and memory	Neuronal transcriptional decoder
*CAMTA2*	Fu-185	Transcriptional decoding	Provides a plausible calmodulin-responsive connection to transcription; direct neuronal Ca^2+^ decoding remains untested	Candidate Ca^2+^-responsive transcriptional decoder
*CREB1*	Mechanistic support only	Transcriptional decoding	Integrates Ca^2+^-responsive kinase and calcineurin–CRTC inputs at activity-regulated genes	Established biochemical decoder
*MEF2C*	Fu-185	Transcriptional decoding	Converts activity-dependent kinase and phosphatase input into neuronal transcription	Core transcriptional decoder; Level 2–3 bridge

Evidence categories: Fu-185, membership in the primary ASC-derived 185-gene set; complementary ASD/NDD, other published autism or neurodevelopmental-disorder genetic evidence; mechanistic support only, functional or interaction evidence without qualifying genetic support. Functional groupings are identified in words and separated by horizontal rules. Fu-185 membership records genetic association and does not by itself establish a direct signal-to-memory mechanism.

**Table 3:** Level 3 transcriptional and chromatin memory. Direct activity-to-chromatin bridges are separated from broader candidate executors and stabilizers. Candidate placement does not assert direct regulation by IP_3_R-derived Ca^2+^.

Gene	Evidence Category	Functional Category	Principal S2M Function	Mechanistic Placement
*CREBBP*	Fu-185	Direct activity-to-chromatin bridge	CREB/other activity-responsive transcription factors recruit CBP lysine acetyltransferase	Direct coactivator bridge
*EP300*	Complementary ASD/NDD	Direct activity-to-chromatin bridge	Activity-responsive transcription-factor recruitment coupled to protein and histone acetylation	Direct coactivator bridge
*KDM6B*	Fu-185	Direct activity-to-chromatin bridge	Neuronal activity can recruit H3K27me3 demethylation to activity-regulated genes	Direct activity-responsive chromatin bridge
*ASH1L*	Fu-185	Histone and DNA-state machinery	Histone methyltransferase shaping developmental transcriptional states	Candidate stabilizer; no direct IP_3_R/Ca^2+^ route established
*EHMT1*	Fu-185	Histone and DNA-state machinery	H3K9 methylation and durable transcriptional repression	Candidate stabilizer; no direct IP_3_R/Ca^2+^ route established
*KMT5B*	Fu-185	Histone and DNA-state machinery	H4K20 methylation and chromatin-state regulation	Candidate stabilizer; no direct IP_3_R/Ca^2+^ route established
*SETD5*	Fu-185	Histone and DNA-state machinery	Chromatin-associated transcriptional regulation	Candidate stabilizer; molecular placement remains broad
*ARID1B*	Fu-185	Nucleosome remodeling	BAF/SWI–SNF nucleosome-remodeling subunit	Candidate executor of accessibility changes
*CHD2*	Fu-185	Nucleosome remodeling	ATP-dependent nucleosome remodeling and promoter/enhancer accessibility	Activity-responsive candidate within the nucleosome-remodeling module; direct Ca^2+^-specific recruitment is not established
*CHD8*	Fu-185	Nucleosome remodeling	Chromatin remodeling and broad developmental transcriptional control	Candidate executor/stabilizer of IP_3_R coupling n
*ADNP*	Fu-185	Reader and coregulator	Chromatin-associated transcriptional and remodeling complexes	Candidate stabilizer; direct pathway link unestablished
*DNMT3A*	Fu-185	Long-term molecular mark	Establishment of DNA methylation patterns	Strong durable-state mechanism; activity-specific route not established
*H3F3A/H3-3A*	Fu-185	Long-term molecular mark	Histone H3.3 incorporation into chromatin	Strong durable-state substrate; activity-specific route not established
*POGZ*	Fu-185	Higher-order chromatin organization	Chromatin regulation and genome organization	Candidate higher-order stabilizer

Evidence categories: Fu-185, membership in the primary ASC-derived 185-gene set; complementary ASD/NDD, other published autism or neurodevelopmental-disorder genetic evidence; mechanistic support only, functional or interaction evidence without qualifying genetic support. Functional groupings are identified in words and separated by horizontal rules. Fu-185 membership records genetic association and does not by itself establish a direct signal-to-memory mechanism.

**Table 4:** Cross-references the 44 retained genes without repeating the functional and mechanistic classifications presented in Table 1, Table 2 and Table 3.

Gene	Level	Evidence Category	Mapped Interaction or Hierarchical Partner(s)
*ATP2B2*	Level 1	Fu-185	CAMK2A (L2), PPP3CA (L2), KCNMA1 (L2)—Ca^2+^ clearance shapes the signal presented to these decoders
*CACNA1A*	Level 1	Complementary ASD/NDD	CAMK2A (L2), PPP3CA (L2), KCNMA1 (L2)—Ca^2+^ entry activates downstream decoders
*CACNA1E*	Level 1	Fu-185	CAMK2A (L2), PPP3CA (L2), KCNMA1 (L2)—Ca^2+^ entry activates downstream decoders
*CACNA2D3*	Level 1	Complementary ASD/NDD	CACNA1A/CACNA1E (L1), CAMK2A/PPP3CA (L2)—regulates Ca^2+^-channel trafficking and signal delivery
*DNM1*	Level 1	Complementary ASD/NDD	SYNJ1 (L1), PPP3CA (L2)—coupled vesicle fission/recycling and calcineurin-sensitive synaptic cycling
*GABBR2*	Level 1	Fu-185	CACNA1A/CACNA1E (L1), KCNMA1 (L2)—GABA-B signaling modulates excitability and Ca^2+^ entry
*GABRB3*	Level 1	Fu-185	SCN1A/SCN2A/KCNQ3 (L1), CACNA1A/CACNA1E (L1)—inhibitory input controls depolarization and Ca^2+^ recruitment
*GRIA2*	Level 1	Fu-185	CACNA1A/CACNA1E (L1), CAMK2A (L2)—excitatory drive recruits Ca^2+^ signaling and plasticity decoding
*GRIN2B*	Level 1	Fu-185	CAMK2A/PPP3CA (L2), CREB1/MEF2C (L2)—NMDA-receptor Ca^2+^ couples synaptic input to decoding and transcription
*KCNQ3*	Level 1	Fu-185	SCN1A/SCN2A (L1), CACNA1A/CACNA1E (L1)—sets the depolarization threshold for activity-dependent Ca^2+^ entry
*SCN1A*	Level 1	Fu-185	GABRB3 (L1), CACNA1A/CACNA1E (L1)—inhibitory-neuron firing controls network recruitment of Ca^2+^ entry
*SCN2A*	Level 1	Fu-185	GRIA2/GRIN2B (L1), CACNA1A/CACNA1E (L1)—action potentials couple excitatory input to Ca^2+^ entry
*SYNJ1*	Level 1	Complementary ASD/NDD	DNM1 (L1), PPP3CA (L2)—phosphoinositide recycling supports repeated synaptic signaling
*ANK2*	Level 1	Fu-185	ITPR1/ITPR3 (L1), ATP2B2 (L1)—organizes membrane/ER signaling domains and Ca^2+^-handling proteins
*HOMER1*	Level 1	Complementary ASD/NDD	SHANK2/SHANK3 (L1), PLCB1 (L1), ITPR1/ITPR3 (L1)—scaffolds receptor-to-IP_3_R signaling
*SHANK2*	Level 1	Fu-185	HOMER1/SHANK3 (L1), GRIN2B/GRIA2/GABBR2 (L1)—organizes postsynaptic receptor and Homer signaling complexes
*SHANK3*	Level 1	Fu-185	HOMER1/SHANK2 (L1), GRIN2B/GRIA2/GABBR2 (L1)—organizes postsynaptic receptor and Homer signaling complexes
*INPP1*	Level 1	Complementary ASD/NDD	PLCB1 (L1), ITPR1/ITPR3 (L1)—phosphoinositide recycling sustains repeated IP_3_ production
*PLCB1*	Level 1	Complementary ASD/NDD	INPP1 (L1), ITPR1/ITPR3 (L1)—generates the IP_3_ ligand for ER Ca^2+^ release
*ITPR1*	Level 1	Complementary ASD/NDD	PLCB1/HOMER1 (L1), CAMK2A/PPP3CA/KCNMA1 (L2), CREB1/MEF2C/CAMTA1/CAMTA2 (L2)—ER Ca^2+^ output feeds decoders
*ITPR3*	Level 1	Fu-185	PLCB1/HOMER1 (L1), CAMK2A/PPP3CA/KCNMA1 (L2), CREB1/MEF2C/CAMTA1/CAMTA2 (L2)—ER Ca^2+^ output feeds decoders
*CAMK2A*	Level 2	Fu-185	GRIN2B/ITPR1/ITPR3 (L1), CREB1/MEF2C/HDAC4 (L2), CREBBP (L3)—phosphorylation-dependent decoding
*PPP3CA*	Level 2	Complementary ASD/NDD	GRIN2B/ITPR1/ITPR3/DNM1/SYNJ1 (L1), HDAC4/MEF2C/CREB1 (L2)—calcineurin-dependent dephosphorylation
*KCNMA1*	Level 2	Fu-185	CACNA1A/CACNA1E/ITPR1/ITPR3 (L1), SCN1A/SCN2A/KCNQ3 (L1)—Ca^2+^-activated electrical feedback
*HDAC4*	Level 2	Fu-185	YWHAG/MEF2C/CAMK2A/PPP3CA (L2), CREBBP/EP300 (L3)—phosphorylation-dependent trafficking and transcriptional repression
*YWHAG*	Level 2	Fu-185	HDAC4 (L2)—14-3-3-dependent phosphoprotein trafficking; isoform specificity remains qualified
*CAMTA1*	Level 2	Complementary ASD/NDD	ITPR1/ITPR3 (L1), CREBBP/EP300 (L3)—proposed Ca^2+^/calmodulin-responsive transcriptional route
*CAMTA2*	Level 2	Fu-185	ITPR1/ITPR3 (L1), CREBBP/EP300 (L3)—candidate Ca^2+^/calmodulin-responsive transcriptional route
*CREB1*	Level 2	Mechanistic support only	CAMK2A/PPP3CA (L2), CREBBP/EP300 (L3)—Ca^2+^-responsive transcription-factor/coactivator coupling
*MEF2C*	Level 2	Fu-185	CAMK2A/PPP3CA/HDAC4 (L2), CREBBP/EP300/KDM6B (L3)—activity-responsive transcription-to-chromatin bridge
*CREBBP*	Level 3	Fu-185	CREB1/MEF2C/HDAC4 (L2), EP300 and the activity-dependent chromatin program (L3)—coactivator recruitment and lysine acetylation
*EP300*	Level 3	Complementary ASD/NDD	CREB1/MEF2C/HDAC4 (L2), CREBBP and the activity-dependent chromatin program (L3)—coactivator recruitment and lysine acetylation
*KDM6B*	Level 3	Fu-185	MEF2C (L2), activity-dependent chromatin program (L3)—removal of H3K27me3 feeds chromatin-state regulation; no direct pairwise complex is implied
*ASH1L*	Level 3	Fu-185	Activity-dependent chromatin program (L3)—candidate executor of histone-state regulation; not a demonstrated direct complex
*EHMT1*	Level 3	Fu-185	Activity-dependent chromatin program (L3)—candidate executor of repressive histone-state regulation; not direct IP_3_R coupling
*KMT5B*	Level 3	Fu-185	Activity-dependent chromatin program, DNMT3A/H3F3A/H3-3A (L3)—convergent durable histone/DNA-state regulation
*SETD5*	Level 3	Fu-185	Activity-dependent chromatin program (L3)—candidate chromatin-associated transcriptional stabilizer
*ARID1B*	Level 3	Fu-185	CHD2/CHD8 (L3), CREBBP/EP300 (L3)—convergent nucleosome accessibility/remodeling
*CHD2*	Level 3	Fu-185	Activity-dependent chromatin program (L3), ARID1B/CHD8 (L3)—candidate activity-responsive nucleosome remodeling and accessibility; no direct physical complex is implied
*CHD8*	Level 3	Fu-185	ARID1B/CHD2 (L3), CREBBP/EP300 (L3)—convergent enhancer/promoter accessibility
*ADNP*	Level 3	Fu-185	ARID1B/CHD8/POGZ (L3)—convergent chromatin-remodeling and regulatory architecture
*DNMT3A*	Level 3	Fu-185	KMT5B/H3F3A/H3-3A (L3), CREBBP/EP300 (L3)—durable DNA/histone-state stabilization
*H3F3A/H3-3A*	Level 3	Fu-185	DNMT3A/KMT5B (L3), CREBBP/EP300 (L3)—histone substrate for durable activity-associated chromatin states
*POGZ*	Level 3	Fu-185	ADNP/ARID1B/CHD8 (L3)—convergent higher-order chromatin regulation

Evidence categories: Fu-185, membership in the primary ASC-derived 185-gene set; complementary ASD/NDD, other published autism or neurodevelopmental-disorder genetic evidence; mechanistic support only, functional or interaction evidence without qualifying genetic support. Functional groupings are identified in words and separated by horizontal rules. Fu-185 membership records genetic association and does not by itself establish a direct signal-to-memory mechanism. Interaction includes a defined biochemical, regulatory, scaffolding, or directional hierarchical connection; it does not necessarily denote direct physical binding. Phrases marked ‘convergent’ identify a shared downstream operation rather than a demonstrated molecular complex.

## Data Availability

No new data were created or analyzed in this study. Data sharing is not applicable to this article.
